# Regulatory T cells in stroke inflammation: Therapeutic perspectives

**DOI:** 10.4103/NRR.NRR-D-24-01424

**Published:** 2025-07-05

**Authors:** Ziyi Sun, Hongyu Zhou, Yongjun Wang, Zixiao Li

**Affiliations:** 1 Department of Neurology, Beijing Tiantan Hospital, Capital Medical University, Beijing, China; 2 China National Clinical Research Center for Neurological Diseases, Beijing, China; 3 National Center for Healthcare Quality Management in Neurological Diseases, Beijing, China; 4 Advanced Innovation Center for Human Brain Protection, Capital Medical University, Beijing, China; 5 Research Unit of Artificial Intelligence in Cerebrovascular Disease, Chinese Academy of Medical Sciences, Beijing, China; 6 Chinese Institute for Brain Research, Beijing, China

**Keywords:** blood–brain barrier, cerebral infarction, immunotherapy, inflammation, interleukin-10, intracerebral hemorrhage, ischemic stroke, regulatory T lymphocytes, stroke rehabilitation, white matter

## Abstract

Regulatory T cells are crucial immunomodulatory cells that play essential roles in both ischemic stroke and intracerebral hemorrhage. These cells are vital in post-stroke inflammation since they suppress immune responses and promote tissue repair. This review thoroughly examines the dynamic changes in the number and function of regulatory T cells and highlights their distinct roles at various stages of stroke progression. In the acute phase (within 5–7 days), regulatory T cells exert neuroprotective effects primarily by reducing inflammation. In the chronic phase (7 days post-onset), these cells support neuroregeneration and functional recovery. The review also explores the emerging role of regulatory T cells in the brain–gut axis, a key mediator of the systemic immune responses following stroke, and discusses its relevance in modulating post-stroke inflammation and repair. Various strategies aimed at enhancing regulatory T cell responses include adoptive transfer of regulatory T cells, administration of pharmacological agents, and induction of mucosal tolerance. All these approaches can potentially enhance the immunomodulatory and repair functions of regulatory T cells. Nevertheless, despite the promising preclinical results, the translation of regulatory T cell–based therapies into clinical practice is associated with challenges related to optimal timing, dosage, and long-term efficacy. Overall, targeting regulatory T cells is a novel and promising immunoregulatory approach for mitigating stroke-induced injury and promoting neural repair.

## Introduction

Stroke, which includes the ischemic stroke (IS) and intracerebral hemorrhage (ICH) subtypes, is the second-leading cause of global mortality and the third-largest contributor to disability-adjusted life years (Krishnamurthi et al., 2020, 2021; Xu et al., 2025). At the core of all stroke pathologies is a metabolic crisis that triggers cascades of neuronal death. In acute ischemic stroke (AIS), this crisis manifests as cerebral hypoperfusion resulting from vascular occlusion, while ICH occurs due to hematoma formation caused by vascular rupture and subsequent secondary injury (Liang et al., 2025; Zheng et al., 2025). Emerging evidence suggests that post-stroke neuroinflammation functions as a dual-edged mechanism, critically modulating the balance between secondary neurodegeneration and reparative processes (Yilmaz et al., 2006; Seifert et al., 2014).

Mechanistically, tissue damage releases damage-associated molecular patterns (DAMPs) that engage pattern recognition receptors, such as Toll-like receptor 4 (TLR4), on microglia and macrophages. This engagement activates nuclear factor kappa-light-chain-enhancer of activated B cells (NF-κB)-driven pro-inflammatory cascades (Anrather and Iadecola, 2016; Shi et al., 2019; Stanzione et al., 2020). Both AIS and ICH exhibit conserved neuroinflammatory signatures characterized by the activation of resident immune cells and the infiltration of peripheral leukocytes. Paradoxically, while this immune response facilitates debris clearance, excessive inflammation—mediated by cytokine storms and oxidative stress—can exacerbate parenchymal damage (Shevach, 2009), indicating the need for precise immunomodulatory interventions.

Cerebral ischemia models have revealed specific temporal thresholds for irreversible injury, with middle cerebral artery occlusion (MCAO) lasting longer than 30 minutes resulting in permanent infarction (Bardutzky et al., 2007; Durukan and Tatlisumak, 2010). In contrast, shorter occlusions (< 15 minutes) resemble transient ischemic attacks (TIAs) (Quenault et al., 2017). This review focuses on studies that use clinically relevant occlusion durations (> 30 minutes), so the mechanisms discussed primarily pertain to the pathophysiology of AIS and ICH.

In this immunological context, regulatory T cells (Tregs)—a subset of CD4^+^ T cells characterized by high expression of cluster of differentiation 25 (CD25) and the transcription factor Foxp3—have emerged as key regulators of post-stroke inflammation (Sun et al., 2023). These cells orchestrate neural repair through dual mechanisms: direct modulation of central nervous system (CNS)–resident microglia and suppression of harmful peripheral immune infiltration (Panduro et al., 2016). Recent studies have highlighted the multifaceted regulatory capabilities of these cells, including the secretion of anti-inflammatory cytokines (such as interleukin-10 [IL-10] and transforming growth factor-beta [TGF-β]), expression of immune checkpoints (such as cytotoxic T lymphocyte–associated protein 4), and metabolic reprogramming of inflammatory microenvironments (Liesz et al., 2009, 2013, 2015; Liesz and Kleinschnita, 2016).

This review explores the spatiotemporal dynamics of Treg-mediated immunomodulation in stroke, focusing on three key objectives: (1) to compare the functions of Tregs in the pathophysiologies of IS and ICH; (2) to evaluate emerging strategies aimed at enhancing Treg efficacy, such as pharmacologic expansion and gut microbiota modulation; and (3) to identify translational barriers and propose solutions for clinical implementation. Importantly, while AIS and ICH differ in their primary injury mechanisms, their convergent DAMP-driven inflammatory networks present opportunities for pan-stroke Treg therapies. By connecting mechanistic insights with therapeutic innovations, this review aims to advance a precision medicine framework for optimizing Treg-targeted interventions, ultimately redefining paradigms in stroke immunotherapy.

## Search Strategy

A computer-based online search of the PubMed database was conducted to retrieve articles published up until December 2024, using the search terms listed in **Additional Table 1**. The results were screened based on titles and abstracts, and studies focusing on the role of Tregs in stroke or their potential as therapeutic strategies were reviewed. Studies without full-text availability were excluded from the analysis. No restrictions were imposed regarding language or study type.

**Additional Table 1:** Search strategy for PubMed database

Query	Search term
#1	“Regulatory T-lymphocytes”
#2	“Ischemic stroke”
#3	“Intracerebral Hemorrhage”
#4	“Stroke rehabilitation
#5	“Immunotherapy”
#6	“Inflammation”
#7	“Blood-brain barrier”
#8	“White matter”
#9	“Interleukin-10”
#10	#1 AND #2
#11	#1 AND #3
#12	#1 AND #4
#13	#1 AND #5
#14	#1 AND #2 AND #3
#15	#1 AND #2 AND #3 AND #4
#16	#1 AND #2 AND #3 AND #5
#17	#1 AND #2 AND #3 AND #6
#18	#1 AND #2 AND #3 AND #7
#19	#1 AND #2 AND #3 AND #8
#20	#1 AND #2 AND #3 AND #9

This table presents our search strategy, with data sourced from PubMed database, covering publications up to December 2024.

## Dynamic Changes and Functional Adaptations of Regulatory T Cells Following Stroke

### Temporal evolution and redistribution of regulatory T cell populations post-stroke

Over the past three decades, substantial evidence from animal studies has demonstrated lymphocyte infiltration into the CNS following IS (Schroeter et al., 1994). Alterations in the dynamic balance between peripheral and central Tregs have been observed in models of IS, particularly after transient MCAO (tMCAO). Early after ischemia, the levels of peripheral blood Tregs significantly decrease, reaching baseline by 72 hours (Kleinschnitz et al., 2013). In contrast, Tregs in the CNS show a progressive accumulation in the ischemic region. During the first week, CD25^+^Foxp3^+^ Tregs constitute less than 5% of the CD4^+^ T cell population in the ischemic core (Gelderblom et al., 2009). However, by days 7 to 10, this proportion increases to 20% (Stubbe et al., 2013), reaching 40% by day 14 and remaining elevated for up to 2 months (Ito et al., 2019b). This spatiotemporal distribution pattern suggests that peripheral Tregs may infiltrate the CNS through remodeling of the blood–brain barrier (BBB). The migration of peripheral Tregs begins with adhesion to the cerebral vasculature between days 1 and 4, followed by infiltration into the brain parenchyma starting on day 5 (Yan et al., 2012; Li et al., 2013b) and continuing until day 28 (Xu et al., 2013). This is further supported by clinical data indicating that in patients with stroke, peripheral blood Treg numbers are at their lowest within 48 hours post-onset (Urra et al., 2009), after which they gradually increase, peaking at around day 21 (Yan et al., 2012). These changes in Treg levels correspond to fluctuations in BBB permeability, with a second peak occurring 6 to 72 hours post-ischemia, suggesting a dynamic interplay between Tregs and BBB integrity (Bernardo-Castro et al., 2020).

In addition to peripheral changes, the dynamics of Tregs may also be influenced by the spleen. Offner et al. (2006b) indicated that while peripheral Tregs remain stable in the early hours following tMCAO, their levels significantly increase at 96 hours, coinciding with spleen atrophy. Despite a reduction in total spleen cell numbers, the percentage of CD3^+^CD4^+^Foxp3^+^ Tregs increases, indicating a degree of resistance to apoptosis or other mechanisms that typically decrease the survival of spleen cells. Spleen volume decreases by approximately 40% within 24–48 hours after permanent MCAO (pMCAO). Normalization of Treg numbers occurs alongside the return of spleen volume to baseline by 96 hours (Seifert et al., 2012). Thus, the spleen may act as a reservoir for Tregs, which subsequently influence immune regulation following a stroke. However, discrepancies between species (mice *versus* rats) and stroke models (tMCAO *versus* pMCAO) complicate direct comparisons, highlighting the need for further investigation in this area.

**Figure 1:**
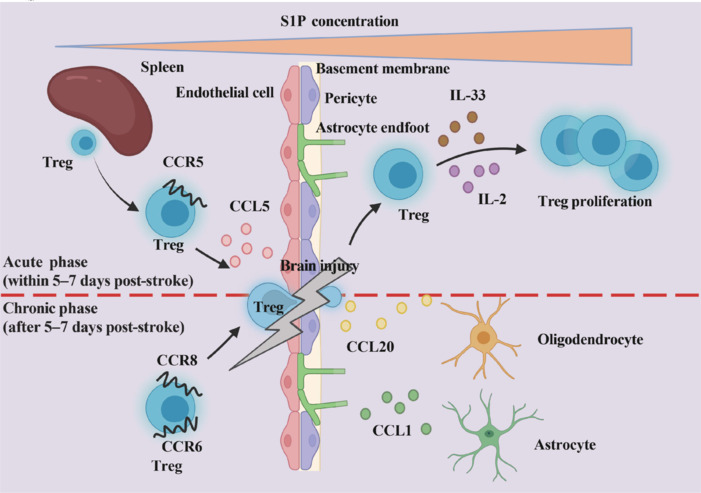
Infiltration, redistribution, and proliferation of regulatory T cells. Peripheral T cells infiltrate the brain during the acute phase of stroke primarily through the CCL5/CCR5 pathway, while the CCL20/CCR8 and CCL1/CCR6 pathways are implicated during the non-acute phase. Upon crossing the blood-brain barrier, peripheral T cells proliferate under the influence of IL-2 and IL-33. Created with BioRender.com. CCL1: C–C motif chemokine ligand 1; CCL5: C–C motif chemokine ligand 5; CCR5: C–C chemokine receptor 5; CCR6: C–C chemokine receptor 6; CCR8: C–C chemokine receptor 8; IL-2: interleukin 2; IL-33: interleukin 33; Treg: regulatory T cell.

The observed expansion of Tregs in the CNS may involve two primary mechanisms: the infiltration of peripheral Tregs and the in situ proliferation of resident CNS Tregs. By days 7 to 14 after ischemia, approximately 60% of CNS Tregs are in a proliferative state, as indicated by Ki-67 positivity, suggesting active division (Stubbe et al., 2013). These proliferating Tregs may originate from peripheral Tregs that have infiltrated the brain or from local Tregs that were previously resident in the CNS. Since the majority of Tregs in the spleen are natural Tregs (Thornton et al., 2010), Tregs entering the brain are predominantly natural Tregs, which undergo activation and proliferation upon entering the CNS. This process is influenced by cytokines in the local environment, particularly interleukin-2 (IL-2) and interleukin-33 (IL-33). IL-33, through its receptor suppression of tumorigenicity 2 (ST2), plays a critical role in Treg proliferation in the ischemic brain, while serotonin stimulation via the 5-hydroxytryptamine receptor 7 (5-HT7) also promotes Treg expansion (Ito et al., 2019b; **Figure 1**). Thus, Treg proliferation in the CNS is a multifaceted process involving both peripheral infiltration and local expansion, with cytokines and receptor interactions serving as key regulatory factors.

Other research suggests that Foxp3^+^ Treg expression in the perihematomal region peaks on day 7 post-ICH, indicating a significant role of Tregs during the acute phase of the injury (Deng et al., 2023). However, conflicting findings have been reported, with some studies showing no significant changes in Treg numbers in the peripheral blood or the hemorrhagic hemisphere on day 3 post-ICH (Lu et al., 2014). These discrepancies may arise from variations in animal models, stroke severity, or experimental methodologies. Beyond animal studies, clinical trials have also yielded differing results. Clinical reports have indicated an increase in peripheral Tregs during the acute phase of ICH, particularly in patients with severe neurological deficits. Elevated IL-10 expression is associated with Treg expansion, and the proportion of Tregs in peripheral blood correlates with disease severity upon hospitalization (Shi et al., 2015). Notably, higher Treg counts at 90 days post-ICH are linked to improved prognosis, underscoring the potential clinical relevance of Treg dynamics in ICH.

In ICH models, the dynamics of Tregs remain less well-defined. A previous study in rats has shown an initial increase in Treg numbers within the brain within 3 days post-ICH, although no significant changes were observed in peripheral blood Tregs (Gao et al., 2014). Other studies have suggested that Foxp3^+^ Treg expression in the perihematomal region peaks on day 7 post-ICH, indicating a significant role of Tregs during the acute phase of injury (Deng et al., 2023). However, conflicting findings have been reported, with some studies noting no significant changes in Treg numbers in either the peripheral blood or the hemorrhagic hemisphere on day 3 post-ICH (Lu et al., 2014). These discrepancies may stem from variations in animal models, stroke severity, or experimental methodologies. In addition to animal studies, clinical trials have also yielded differing results. Clinical reports have indicated an increase in peripheral Tregs during the acute phase of ICH, particularly in patients with severe neurological deficits. Elevated IL-10 expression is associated with Treg expansion, and the proportion of Tregs in peripheral blood correlates with disease severity upon hospitalization (Shi et al., 2015). Notably, higher Treg counts at 90 days post-ICH are linked to improved prognosis, indicating the potential clinical relevance of Treg dynamics in ICH.

In conclusion, while substantial progress has been made in understanding Treg dynamics in IS, the role of Tregs in ICH remains less well-elucidated. Additional studies are essential to clarify these immune cell dynamics and their potential therapeutic implications.

### Molecular and cellular mechanisms governing regulatory T cell recruitment to damaged brain regions following ischemic stroke

The recruitment of Tregs involves complex molecular interactions that are essential for their adhesion and directional migration to target areas. Tregs primarily adhere to and are retained at the target region through lymphocyte function-associated antigen-4 (LFA-4) and intercellular adhesion molecule-1 (ICAM-1), after which they initiate the infiltration process. During the acute phase of stroke (5–7 days following IS), Treg infiltration into the brain may be mediated through several pathways. First, the interaction between CCR5 and CCL5 plays a crucial role in Treg recruitment. After tMCAO, Tregs exhibit early upregulation of CCR5, while CCL5 levels on endothelial cells increase, facilitating the interaction between Tregs and endothelial cells and driving Treg migration toward the inflammatory site (Li et al., 2017). Second, the establishment of a sphingosine-1-phosphate (S1P) gradient is another key factor for Treg migration. S1P creates a gradient between immune cells and areas with higher S1P concentrations, directing Tregs to migrate toward ischemic regions and enhancing their positioning at inflammatory or damaged sites (Matloubian et al., 2004). Specifically, at 24 hours post-MCAO, the S1P concentration is the lowest in the spleen, moderate in the blood, and highest in the ischemic core region, forming a significant gradient. Thus, Tregs may migrate from the spleen to peripheral regions on the basis of this gradient and ultimately accumulate in the ischemic core (Lucaciu et al., 2020; **Figure 1**).

Additionally, the involvement of CCR4 and its ligands is notable. CCR4 promotes Treg migration to the brain by binding to CCL17 and CCL22, which are secreted by activated microglia. This interaction helps reduce inflammation and supports brain recovery (Lee et al., 2024). Furthermore, brain dendritic cells play a crucial role in Treg infiltration. In the acute phase following tMCAO, brain dendritic cells gradually increase in number and, through upregulation of major histocompatibility complex (MHC) II and CD80, activate T cells and maintain Treg function in the ischemic region. This process further promotes Treg recruitment and activation (Felger et al., 2010). Taken together, these mechanisms enable Tregs to effectively migrate to the damaged brain area, contributing to immune modulation during stroke-induced inflammation. In the chronic phase of stroke (5–7 days post-IS), Tregs show significantly increased expression of C–C chemokine receptors 8 and 6, which correlates with elevated expression of C–C chemokine ligand 1 on astrocytes and C–C chemokine ligand 20 on oligodendrocytes in the ischemic brain, thereby promoting Treg infiltration and activity in the damaged region (Ito et al., 2019b; Zhang et al., 2021a; **Figure 1**).

Notably, Treg infiltration in the brain is influenced by both age and sex. Aged mice exhibit a higher number of Tregs in the brain on day 15 post-stroke, with male mice showing a higher count than female mice. Thus, demographic factors such as age and sex modulate the Treg response to ischemic injury (Ahnstedt et al., 2020).

### Functional plasticity and adaptive responses of regulatory T cells in stroke pathophysiology

Tregs undergo major functional changes after a stroke. Recent research has shown that the gene expression profiles of brain and spinal cord Tregs in IS and experimental autoimmune encephalomyelitis mice are more similar to each other than to those of Tregs found in the spleen. Gene ontology enrichment analyses have suggested that the characteristics of Tregs are primarily shaped by the local tissue microenvironment rather than the underlying pathological conditions or antigen specificity. Thus, within the CNS, Tregs undergo functional adaptation, differentiating them from peripheral Tregs and potentially contributing to the maintenance of immune homeostasis in neuroinflammatory environments (Watanabe et al., 2024). Post-stroke brain Tregs exhibit elevated expression of programmed cell death protein 1 (PD-1) and reduced expression of CD25, which may play a critical role in sustaining long-term immune tolerance to CNS antigens (Schulze et al., 2021). In contrast to conditions such as multiple sclerosis, where impaired PD-1 expression and elevated CD25 expression on T cells lead to autoimmunity (Bellamy et al., 1985; Khoury et al., 2000; Pittet et al., 2011), patients with stroke show strong PD-1 expression without an increase in CD25 levels. This unique post-stroke expression pattern may serve as a protective mechanism to prevent inflammatory autoimmunity.

**Figure 2:**
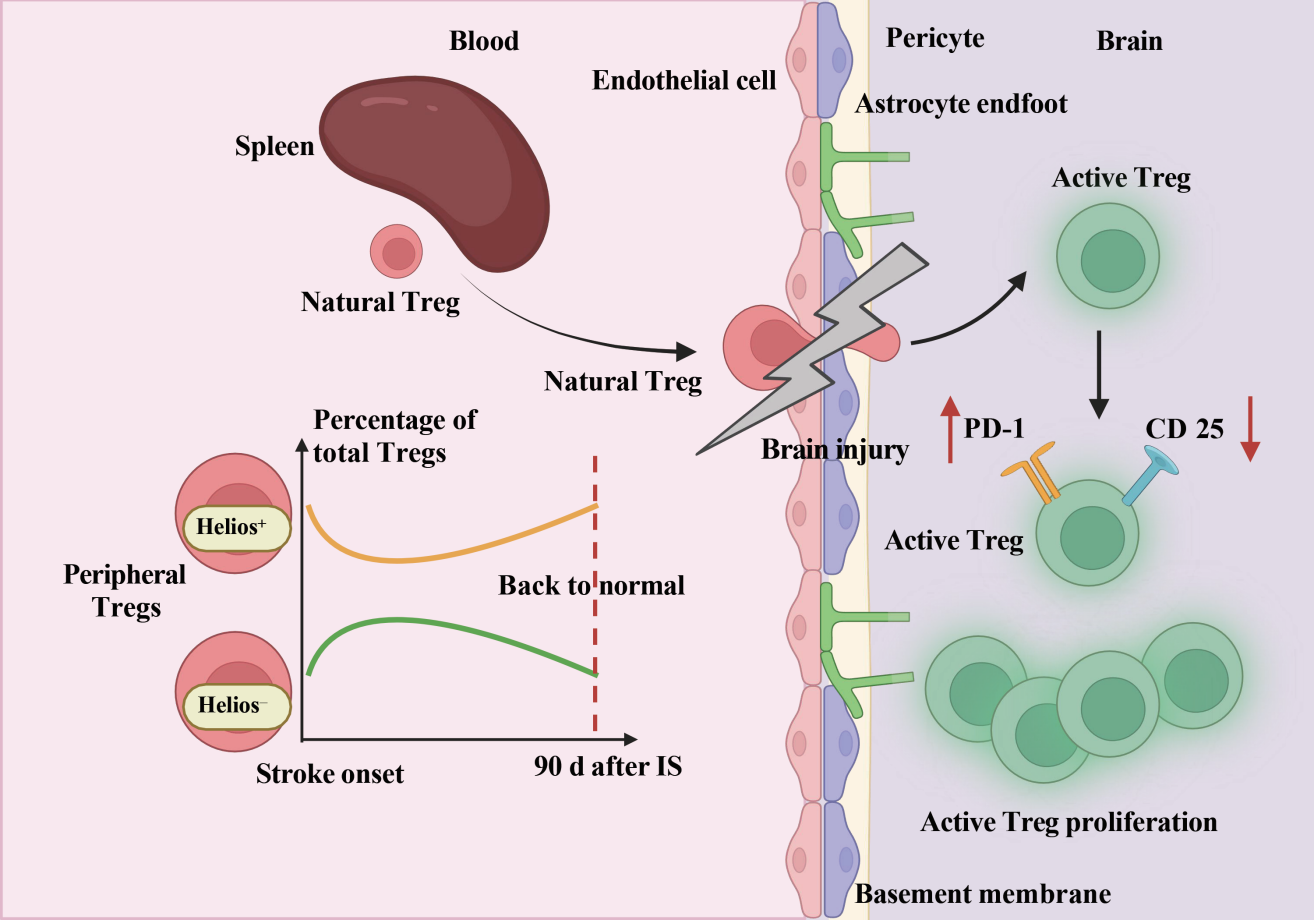
Alterations in Treg function. Peripheral Tregs remain inactive until they enter the brain, where they become activated. After activation, these Tregs exhibit elevated PD-1 expression and reduced CD25 expression, potentially mitigating the risk of autoimmunity. Additionally, the transcription factor Helios undergoes significant changes in peripheral T cells following stroke, leading to a reduction in the proportion of H^+^ cells and an increase in H^–^ cells. This altered balance gradually returns to a more moderate level approximately 90 days (d) after stroke. Created with BioRender.com. CD25: Cluster of differentiation 25; Helios: a transcription factor in Tregs; H^–^: Helios-negative cells; H^+^: Helios-positive cells; PD-1: programmed death-1; Tregs: regulatory T cells.

Helios, a transcription factor from the Ikaros family, was first identified in Tregs in the late 1990s. Under physiological conditions, Helios-positive (H^+^) Tregs constitute up to 70%–90% of the total Treg population (Zabransky et al., 2012). H^+^ and Helios-negative (H^–^) Tregs show substantial differences, with approximately 1000 genes differing between these subsets (Zabransky et al., 2012). H^+^ Tregs are characterized by the presence of antigens typically found on activated, suppressive Tregs, whereas H^–^ Tregs are more likely to secrete interferon-γ (IFN-γ), indicating a pro-inflammatory role. These differences suggest that H^+^ Tregs primarily function as suppressive cells, while H^–^ Tregs may contribute to inflammatory responses (Getnet et al., 2010; Thornton et al., 2019; **Figure 2**).

Lukasik et al. (2023) examined the peripheral Treg population in patients with stroke and found that on the first day after stroke, the ratio of H^+^ to H^–^ Tregs shifted toward the pro-inflammatory H^–^ subtype. This shift persisted for at least 10 days and partially recovered by day 90. Interestingly, on day 3, a higher proportion of H^+^ Tregs was associated with an increased risk of stroke-related infections and higher National Institutes of Health Stroke Scale scores, although it could not independently predict stroke recovery outcomes. This dynamic shift in the balance between H^+^ and H^–^ Tregs highlights a systemic alteration in the balance between suppressive and inflammatory processes following stroke. Further research is needed to explore the underlying causes and implications of this shift. The role of Helios in maintaining Treg stability and function, particularly the mechanisms regulating Helios expression, remains incompletely understood and is a critical area for ongoing investigation (Kim et al., 2015; Chougnet and Hildeman, 2016).

## Peripheral Regulatory T Cells in Acute Stroke: Neurovascular Protection and Immune Modulation (Within 5–7 Days Post-Stroke)

Although Tregs are predominantly recruited to the brain during the later stages of stroke, accumulating evidence from Treg-depletion and therapeutic studies indicates that they modulate stroke outcomes in the early days following ischemia (Xie et al., 2014; Zhang et al., 2018a). Thus, Tregs may influence stroke pathophysiology early on by regulating the peripheral immune system before their substantial infiltration into the brain. Subsequent experimental studies involving either antibody-mediated Treg depletion or inducible Foxp3-knockout mice treated 3 days after MCAO failed to show significant effects on stroke outcomes (Liesz et al., 2013; Stubbe et al., 2013). Thus, while the precise anatomical site of Treg action post-stroke remains undefined, current evidence emphasizes their critical immunomodulatory role during the early phase of stroke, particularly within the first 3 days; this role is likely mediated by peripheral mechanisms. These mechanisms may involve suppressing effector T cell activation, limiting the clonal expansion of autoantigen-specific T cells, and regulating the transendothelial migration of effector T cells through as-yet unidentified pathways. Furthermore, Treg depletion initiated 7 days after stroke does not alter infarct volume or tissue loss at later stages but significantly exacerbates neurological deficits and impairs functional recovery (Saino et al., 2010; Ito et al., 2019b; Shi et al., 2021), highlighting the indispensable role of Tregs in neuronal repair during the chronic phase of stroke recovery. Notably, the mechanisms underlying Treg-mediated regulation in IS and ICH are largely conserved, with only subtle differences in their modulation of effector T cell responses.

### Role of regulatory T cells in maintaining blood–brain barrier integrity and reducing vascular leakage

The BBB is a highly intricate and dynamic microvascular structure that plays a crucial role in maintaining CNS homeostasis by selectively regulating the influx of essential molecules and the efflux of waste products between the blood and the brain (Daneman and Prat, 2015). Its central structure is the neurovascular unit, which consists of brain microvascular endothelial cells surrounded by astrocytic end-feet, pericytes, and the basal membrane (Persidsky et al., 2006). This highly regulated barrier is critical to the pathophysiology of stroke, and BBB dysfunction varies depending on the severity and duration of the ischemic event. Research has shown that the permeability of the BBB follows a biphasic pattern during the four major stages of stroke (Bernardo-Castro et al., 2020), with Tregs playing a key role in maintaining BBB integrity, particularly during the second and third stages of stroke.

In the hyperacute phase (within the first 6 hours of stroke onset), the BBB is initially compromised, which is characterized by hypoxia and glucose deprivation. These conditions lead to the accumulation of Na^+^ and Ca^2+^ in the cells of the neurovascular unit (e.g., astrocytes and brain microvascular endothelial cells). This ion accumulation triggers glutamate excitotoxicity (Liebeskind et al., 2019), oxidative stress injury, and mitochondrial dysfunction (Bernardo-Castro et al., 2020). Additionally, the upregulation of matrix metalloproteinases (MMPs), particularly MMP-2, degrades tight junction proteins and components of the basal membrane, resulting in BBB leakage (Yang and Rosenberg, 2011).

**Figure 3:**
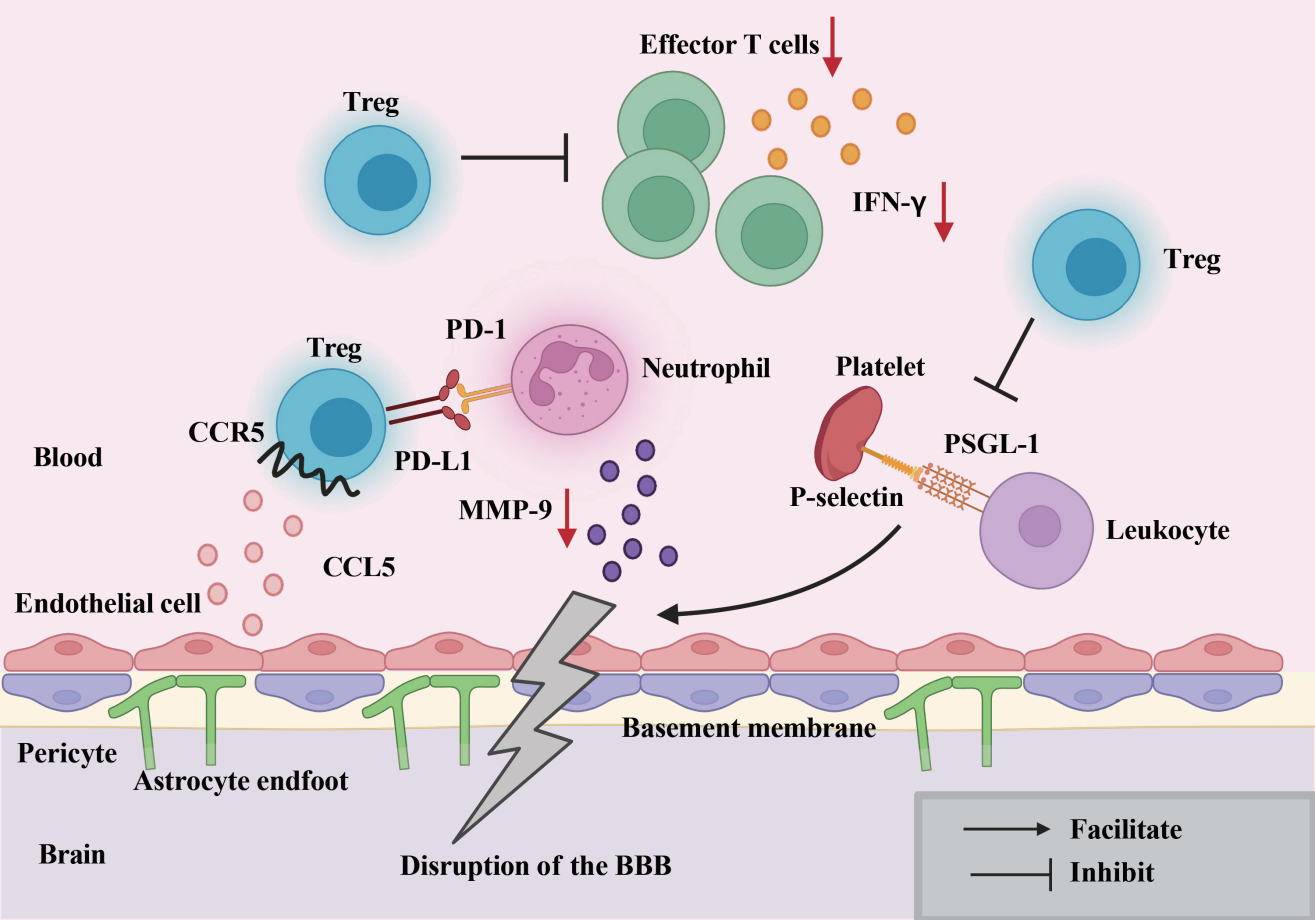
Mechanism of peripheral Tregs in the early stage (within 5–7 days post-stroke). During this early stage, Tregs infiltrate the ischemic tissue via the CCR5/CCL5 pathway, reducing neutrophil-induced MMP-9 levels through the PD-1/PD-L1 pathway, thereby preserving the integrity of the BBB. The upregulation of CCR5 expression in Tregs enhances PD-L1 expression, further inhibiting MMP-9. Additionally, Tregs can prevent platelet binding to PSGL-1 on lymphocytes via P-selectin, inhibiting platelet-leukocyte aggregate formation and protecting BBB integrity. Furthermore, Tregs suppress effector T cell activity, including their secretion of pro-inflammatory cytokines such as IFN-γ. Created with BioRender.com. BBB: Blood–brain barrier; CCL5: C–C motif chemokine ligand 5; CCR5: C–C chemokine receptor type 5; IFN-γ: interferon gamma; MMP-9: matrix metalloproteinase 9; PD-1: programmed death-1; PD-L1: programmed death ligand 1; PSGL-1: P-selectin glycoprotein ligand-1; Tregs: regulatory T cells.

The hyperacute phase is followed by the acute phase, lasting from 6 to 72–96 hours, during which BBB permeability peaks a second time and immune cells begin to play a crucial role. Neutrophils, as the major component of peripheral immune cells (Price et al., 2004), secrete excessive reactive oxygen species (Krizbai et al., 2005), along with a range of inflammatory factors (e.g., interleukin-1 beta [IL-1β], interleukin-6 [IL-6], tumor necrosis factor-alpha [TNF-α]) and chemokines (e.g., CCL2, CCL3, CCL5) (Jickling et al., 2015). These factors directly damage the BBB and also exacerbate the damage by inducing MMP-9 expression (Li et al., 2013b; Li and Chen, 2023). Tregs help inhibit the excessive expression of MMP-9 through direct cell-cell contact with neutrophils, thereby reducing proteolytic damage to the BBB and providing neuroprotection. This mechanism depends on the interaction between programmed death-ligand 1 (PD-L1) on Tregs and PD-1 on neutrophils. Blocking this interaction with neutralizing antibodies against PD-L1 or PD-1 eliminates the inhibition of MMP-9 by Tregs (Li et al., 2013b, 2014a). Furthermore, intravenous administration of Tregs has been shown to reduce BBB damage, although Tregs were not observed in the brain parenchyma, suggesting that peripheral Tregs play a crucial role in protecting the BBB by inhibiting MMP-9 production and reducing leukocyte infiltration and brain edema (Li et al., 2013b; **Figure 3**).

The subacute phase (approximately 1 week post-stroke) marks the gradual recovery of the BBB, with a shift from a pro-inflammatory to an anti-inflammatory immune response. During this phase, monocytes and glial cells secrete cytokines (e.g., interleukin-10 [IL-10], interleukin-4 [IL-4]) and neurotrophic factors that help reduce inflammation and promote tissue repair (Rui et al., 2017). Tregs play a pivotal role in this process by promoting resolution of inflammation and enhancing BBB repair through regulation of the immune response.

In the chronic phase (around 6 weeks post-stroke), although some BBB damage persists, its function has been largely restored. Tight junctions are reestablished; new junctional proteins are synthesized; and the stability of the brain’s endothelial layer is reinforced. Meanwhile, immune cells further transition to an anti-inflammatory phenotype, ensuring long-term BBB stability. Tregs continue to play an essential role in maintaining BBB integrity and promoting the repair of the neurovascular unit by regulating the immune response (Shindo et al., 2016).

Thus, Tregs not only protect the BBB by modulating immune cell interactions during stroke but also play a vital neuroprotective role by activating repair mechanisms for the BBB. Through their effects in reducing inflammation, inhibiting excessive immune cell activation, and promoting BBB repair and stability, Tregs are indispensable immune regulators in stroke neuroprotection.

### Prevention of platelet-leukocyte aggregation and microthrombosis by regulatory T cells

Leukocytes are recruited into brain tissue following human and experimental stroke (Akopov et al., 1996; Price et al., 2004). The breakdown of the BBB facilitates the infiltration of peripheral leukocytes into the injured brain. In a cyclical manner, these leukocytes exacerbate BBB disruption by releasing pro-inflammatory cytokines, ROS, and MMPs (Dokalis and Prinz, 2019; Shi et al., 2019). After leukocyte accumulation, platelets aggregate in the post-ischemic cerebral microvasculature (Tailor et al., 2005). This process is mediated by the interaction of platelet-associated P-selectin with its ligand P-selectin glycoprotein ligand-1 (PSGL-1) on leukocytes (Ishikawa et al., 2003, 2004). Tregs effectively inhibit circulating platelet-leukocyte aggregate formation in peripheral blood, thereby safeguarding BBB integrity (**Figure 3**).

### Chemokine-mediated tethering and retention of regulatory T cells at ischemic sites for endothelial protection

CCR5 is a chemokine receptor predominantly expressed on T cells that plays a pivotal role in the recruitment and activation of inflammatory cells. Under certain pathological conditions, CCR5 is preferentially expressed on Tregs, contributing to various immune functions (Yurchenko et al., 2006). Lin et al. (2023) reported that after ICH, CCR5 expression was significantly upregulated in both the brain and serum. This increase may exacerbate BBB disruption through the Janus kinase 2 (JAK2)/signal transducer and activator of transcription 3 (STAT3) signaling pathway, thereby intensifying neuroinflammation and brain edema. These findings suggest that CCR5 upregulation could be a detrimental factor in BBB damage.

In contrast, Li et al. (2017) demonstrated that the injection of CCR5^–/–^ Tregs into stroke mice did not improve BBB integrity or neurological function, highlighting the crucial role of CCR5 in Treg-mediated BBB protection. These findings underscore the dual role of CCR5: although its upregulation may be harmful in some contexts, under the influence of Tregs, CCR5 becomes essential for BBB protection, particularly in neuroinflammatory conditions. Furthermore, after ICH, CCL5 expression is significantly upregulated in both the brain and peripheral tissues. Administration of recombinant CCL5 (rCCL5) further exacerbates BBB disruption (Lin et al., 2023). Upon activation, Tregs engage CCR5, which binds to its ligand CCL5. This interaction enhances the contact between Tregs and damaged endothelial cells, limiting the infiltration of other immune cells and preserving BBB integrity. Although upregulation of CCR5 and CCL5 may have potentially harmful effects, Tregs mitigate these effects through receptor-ligand interactions, protecting the BBB by reducing immune cell infiltration. Moreover, the induction of CCR5 on Tregs also leads to the upregulation of PD-L1 expression, further enhancing the suppressive effects of Tregs on MMP-9 (Li et al., 2017; **Figure 3**).

### Suppression of effector T cell activation to modulate peripheral and central inflammatory responses

Tregs play a pivotal role in modulating the activation of effector T cells, particularly in the context of stroke, where their immunoregulatory function is crucial for maintaining the stability of the BBB (Liesz et al., 2011). After a stroke, effector T cells contribute to neuroinflammation by promoting the release of pro-inflammatory cytokines, enhancing oxidative stress, and directly compromising BBB integrity. Tregs counteract these effects by suppressing the aberrant activation of effector T cells, thereby reducing inflammation and preserving BBB function.

In ICH, effector T cells, particularly T helper 17 (Th17) cells, exacerbate BBB disruption by upregulating the expression of interleukin-17A (IL-17A). IL-17A induces the secretion of pro-inflammatory cytokines and amplifies oxidative stress (Liesz et al., 2011). IL-17A, a key member of the interleukin-17 (IL-17) cytokine family, is primarily secreted by Th17 cells. Upon activation, it stimulates the production of reactive oxygen species and reduces the expression of tight junction proteins, leading to BBB compromise (Huppert et al., 2010). Additionally, Shi et al. (2022) reported that IL-17A binds to interleukin-17 receptor C (IL-17RC) receptors on endothelial cells and pericytes, directly affecting the vascular wall to further exacerbate endothelial cell injury and accelerate BBB breakdown. Maintaining a homeostatic balance between IL-17-producing cells (e.g., Th17 cells) and Tregs is essential for mitigating neuroinflammatory responses and preserving BBB integrity (Hu et al., 2014; Noh et al., 2018; Cui et al., 2021). Tregs exert protective effects by suppressing the activation of Th17 and other effector T cells, thereby reducing pro-inflammatory cytokine release and attenuating BBB damage (Liesz et al., 2015).

In IS, the primary role of Tregs is to suppress effector T cell activation and, consequently, mitigate inflammation. Specifically, Tregs downregulate IFN-γ expression, thereby reducing its pro-inflammatory effects and alleviating BBB disruption (Liesz et al., 2015). An *in vitro* study showed that splenic Tregs significantly inhibit the activation of effector T cells and decrease IFN-γ production following stroke (Liesz et al., 2013). Furthermore, by the 50th day post-stroke, Tregs continue to suppress the activation of infiltrating effector T cells within the brain and reduce IFN-γ release (Liesz et al., 2009).

Thus, in both ICH and IS, Tregs confer neuroprotection by suppressing effector T cell activation and thereby preserving BBB integrity, although their mechanisms of action differ in the two conditions. In ICH, Tregs primarily modulate Th17 cells and IL-17A signaling pathways to reduce BBB damage and brain edema. In contrast, in IS, Tregs attenuate IFN-γ-mediated pro-inflammatory responses, thereby alleviating neuroinflammation (**Figure 3**). These findings suggest that targeting Treg-mediated immune regulation could serve as a novel therapeutic strategy for stroke treatment, warranting further investigation.

## Brain-Resident Regulatory T Cells in Subacute and Chronic Stroke: Neurorepair and Functional Recovery (After 5–7 Days Post-Stroke)

### Regulatory T cells enhance neurogenesis and neuronal circuit plasticity via interleukin-10

IL-10 is a critical anti-inflammatory cytokine that plays a key role in modulating the inflammatory response through Tregs. In both IS and ICH, Tregs exert their anti-inflammatory effects by secreting IL-10, which helps mitigate neural damage and promotes tissue repair.

**Figure 4:**
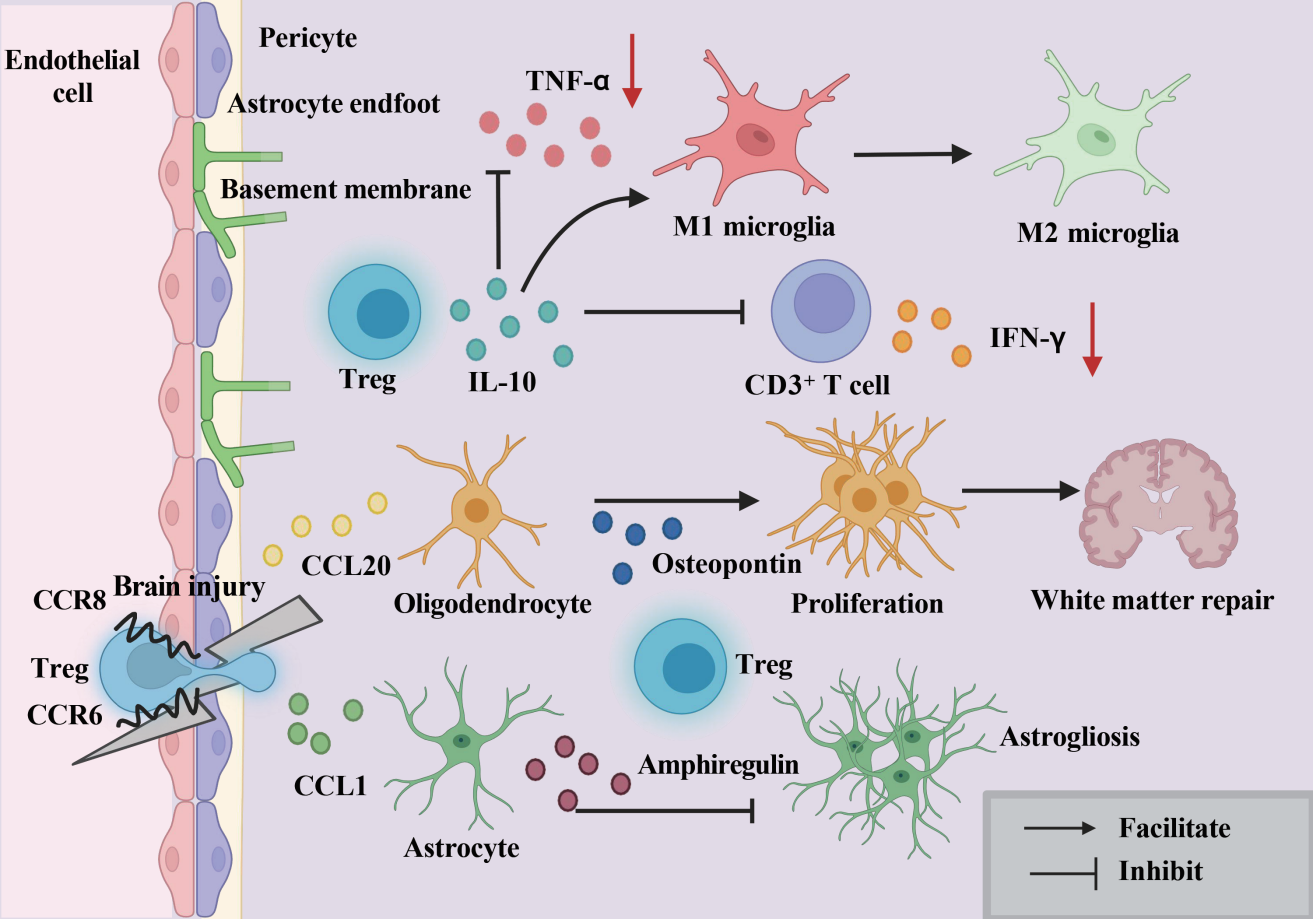
Mechanism of brain Tregs in the non-early stages of stroke (after 5–7 days post-stroke). In the later stages, Tregs infiltrate the brain via the CCR6/CCL20 and CCR8/CCL1 pathways. Treg-induced IL-10 suppresses pro-inflammatory cytokines, such as TNF-α and IFN-γ. Tregs also promote the polarization of microglia towards the M2 subtype. Additionally, Tregs stimulate oligodendrocyte proliferation, aiding in white matter repair. They help curb excessive astrogliosis, thereby facilitating neurorepair. Created with BioRender.com. CCL1: C–C motif chemokine ligand 1; CCL20: C–C motif chemokine ligand 20; CCR6: C–C Chemokine receptor type 6; CCR8: C–C chemokine receptor type 8; IL-10: interleukin-10; IFN-γ: interferon gamma; M2: macrophage subtype 2; Tregs: regulatory T cells; TNF-α: tumor necrosis factor alpha.

In the context of IS models, IL-10 therapy has demonstrated significant anti-inflammatory and neurorepair effects in experimental models of cerebral ischemia. Liesz et al. (2014) reported that IL-10 reduces the infarct size following ischemia, with notable upregulation of its expression between days 3 and 7 post-stroke. IL-10 helps minimize secondary injury by downregulating the expression of neurotoxic inflammatory mediators, including TNF-α and IFN-γ. Additionally, IL-10 promotes neurogenesis by binding to the IL-10 receptor (IL-10R) on neural stem cells (NSCs), subsequently activating the extracellular signal-regulated kinaseand STAT3 pathways (Pereira et al., 2015; **Figure 4**).

In ICH models, IL-10 expression is notably downregulated in peripheral blood and brain tissue surrounding the hematoma. However, overexpression of IL-10 can significantly attenuate the inflammatory response following ICH (Laffer et al., 2019; Song et al., 2019). In a study by Gao et al. (2014), transplanted NSCs were shown to increase Treg populations and promote the expression of anti-inflammatory cytokines, such as IL-10 and TGF-β, while reducing the expression of pro-inflammatory cytokines such as IL-6. This action helps mitigate brain tissue damage, further illustrating the anti-inflammatory role of Tregs through IL-10 secretion following ICH. Additionally, IL-10 plays a key role in promoting NSC proliferation and differentiation. In another study, Gao et al. (2022) found that IL-10-modified mesenchymal stromal cells significantly enhanced Treg numbers, reduced glial fibrillary acidic protein (GFAP) expression, increased the number of neurofilament-positive cells, and promoted the regeneration of neurons and axons after spinal cord injury. These findings indicate that IL-10 alleviates inflammation and also contributes to neurorepair under inflammatory conditions.

In addition to Tregs, IL-10-producing regulatory B cells (B10 cells), which also secrete IL-10, have been shown to significantly improve stroke outcomes. B10 cells suppress pro-inflammatory responses, enhance Treg populations, and reduce infarct volume, underscoring the critical role of IL-10 in neuroprotection (Bodhankar et al., 2013, 2014a, b). These cells support brain recovery by enhancing immune regulation, further highlighting the importance of IL-10 in promoting tissue repair and recovery.

### Microglial polarization and white matter repair mediated by regulatory T cells

Microglial cells are critical immune cells in the brain that respond rapidly after stroke, playing essential roles in both hemorrhagic and ischemic strokes (Chen et al., 2025; Li et al., 2025). In IS, microglial cells begin to activate within minutes of ischemia and reach peak activity by day 10 (Schilling et al., 2005). In brain hemorrhage, microglial cells are considered the first non-neuronal cells to respond to ICH injury (Guo et al., 2022). The interaction between microglial cells and Tregs is similar in both IS and brain hemorrhage. This interaction not only mitigates inflammation but also promotes neural repair.

Microglial cells are activated following a stroke, and this activation is categorized into two types: classical (M1) and alternative (M2) (Bonsack et al., 2016). M1 microglial cells exacerbate neuronal injury by releasing pro-inflammatory cytokines and neurotoxic substances, while M2 microglial cells facilitate reparative anti-inflammatory responses (Zeng et al., 2022). Two studies have suggested that the neuroinflammation following a stroke is a complex and persistent process rather than a simple bifurcation (Chiu et al., 2013; Lewis et al., 2014). Nonetheless, the M1/M2 classification remains widely used. Interactions between M1 microglial cells and T helper 1 (Th1) or Th17 cells have been shown to correlate with brain injury, while interactions between M2 microglial cells and T helper 2 (Th2) or Tregs have been shown to support recovery (Wang et al., 2016; Straeten et al., 2024). M1 microglial cells increase the expression of hypoxia-inducible factor 1αin Tregs, which triggers sirtuin 2 (Sirt2), inhibiting Foxp3 expression and reducing the anti-inflammatory capacity of Tregs. As a result, M1 microglial cells suppress the regulatory function of Tregs (Shu et al., 2019). In contrast, M2 microglial cells promote Treg differentiation by secreting IL-10 and TGF-β, enhancing anti-inflammatory effects, and mitigating brain tissue damage (Brea et al., 2014).

IL-10 released by Tregs downregulates the secretion of pro-inflammatory cytokines such as TNF-α and IL-1β from microglial cells (Yang et al., 2014) and upregulates glycogen synthase kinase 3β in microglial cells. Glycogen synthase kinase 3βphosphorylates and inactivates phosphatase and tensin homolog, which shifts microglial cells toward the M2 phenotype, effectively alleviating the inflammation induced by IS (Xie et al., 2015; Zhou et al., 2017). Additionally, Tregs reduce microglial inflammation by inhibiting the STAT3 signaling pathway and its transcriptional activation. Tregs also influence microglial gene expression related to immune cell recruitment and homeostasis, although this aspect requires elucidation (Benakis et al., 2022; **Figure 4**).

In conclusion, Tregs promote the differentiation of microglial cells into the M2 phenotype following a stroke, which, in turn, induces Treg differentiation and reinforces anti-inflammatory responses.

Additionally, Shi et al. (2021) proposed that osteopontin secreted by Tregs enhances neurorepair by binding to integrin receptors on microglial cells, which increases the number of oligodendrocytes involved in repairing white matter. However, the findings did not clarify whether this increase is a result of differentiation from oligodendrocyte precursor cells (OPCs) (Zera and Buckwalter, 2021).

Furthermore, IL-10 released by Tregs may influence oligodendrocyte maturation. In a mouse model, the depletion of Tregs significantly reduced the number of mature oligodendrocytes, indicating their role in oligodendrocyte differentiation (Dombrowski et al., 2017). The platelet-derived growth factor receptor α (PDGFRα), a key marker of OPCs, is regulated by the transcription factor Olig2, which is essential for OPC differentiation and myelin repair (Zou et al., 2023). Recent studies have shown that Tregs regulate the OPC microenvironment through IL-10 signaling, promoting both anti-inflammatory and pro-differentiation factors. IL-10 signaling not only inhibits inflammation but also enhances the maturation of PDGFRα^+^ OPCs. While the precise mechanisms remain to be fully elucidated, IL-10 has been shown to influence OPC activity and differentiation through specific receptors and signaling pathways. *In vitro* experiments using lipid nanoparticles to deliver Olig2 mRNA to OPCs demonstrated efficient differentiation from OPCs to oligodendrocytes. In an ischemic stroke mouse model, Olig2 mRNA delivery improved BBB integrity, facilitated remyelination, and supported cognitive recovery. RNA sequencing analyses revealed enrichment of processes associated with learning and memory, underscoring the therapeutic potential of mRNA-based approaches for neural regeneration. These findings suggest that Treg-derived IL-10 may regulate Olig2 expression, thereby promoting OPC maturation and repair. Additional studies are required to clarify the precise molecular mechanisms underlying this process (Xu et al., 2024).

### Astrocyte modulation by regulatory T cells to prevent reactive gliosis and secondary damage

Astrocytes are the most abundant cell type in the CNS, constituting approximately one-third of all cells in this region. They play a central role in the acute response to brain injury (Wirenfeldt et al., 2011). Under normal physiological conditions, astrocytes support neuronal metabolism, facilitate synapse formation, and provide neuroprotection. However, after acute neurological damage, such as IS, astrocytes undergo phenotypic transformation, differentiating into two distinct functional phenotypes: A1 and A2 (Liddelow et al., 2017). A1 astrocytes, which are induced by neuroinflammation and ischemia, exhibit pro-inflammatory properties and neurotoxicity, impairing neuronal survival. These cells are characterized by increased proliferation, morphological enlargement, elevated expression of GFAP, and a tendency to form glial scars that hinder neuronal repair (Ito et al., 2019b). In contrast, A2 astrocytes possess anti-inflammatory properties and actively contribute to the repair process.

Tregs play a critical role in the repair process following neural injury, particularly in modulating astrocyte activation. Research has shown that depletion of Tregs leads to increased activation of astrocytes, resulting in heightened expression of pro-inflammatory factors and exacerbating neuronal damage. In the tMCAO mouse model, Treg depletion resulted in excessive activation of astrocytes and an intensified inflammatory response. Tregs modulate A1 astrocytes through secretion of amphiregulin (AREG), which suppresses their proliferative behavior, and by downregulating the IL-6-STAT3 signaling pathway, which inhibits the expression of GFAP in these cells. This process effectively reduces the proliferation of neurotoxic A1 astrocytes, thereby promoting neural repair and regeneration (Beurel et al., 2014; Ito et al., 2019b). Furthermore, astrocytes help maintain stable expression of Foxp3^+^ Tregs through the IL-2/signal transducer and activator of transcription 5 (STAT5) signaling pathway (Xie et al., 2015). This interaction supports the biological functions of Tregs and enhances the progression of neural repair (**Figure 4**).

In conclusion, the interaction between astrocytes and Tregs is essential for restoring neurological function after a stroke. Astrocytes not only facilitate the repair process following neurological injury but also promote recovery by regulating Treg activity and enhancing neuroprotection.

## Potential Adverse Effects of Regulatory T Cells

None of the existing studies have demonstrated a detrimental role of Tregs in ICH. However, in IS, their functional mechanisms appear to be more complex, exhibiting both protective and potentially harmful effects under certain conditions.

Kleinschnitz et al. (2013) were the first to report that within 24 hours post-tMCAO, Tregs interact with endothelial cells in the ischemic brain through the LFA-1/ICAM-1 signaling pathway, leading to microvascular dysfunction. This detrimental effect is platelet-dependent, since Treg-platelet interactions result in reduced cerebral blood flow, increased fibrinogen deposition in brain tissue, and, ultimately, larger infarct volumes and thrombus formation. Subsequently, Schuhmann et al. (2015) corroborated these findings, showing that elevated Treg numbers exacerbate thromboinflammation in transient stroke models, thereby worsening stroke outcomes. In contrast, Na et al. (2015) presented conflicting evidence, demonstrating that enhancement of Treg activity improves stroke outcomes in both permanent and transient mechanical vascular occlusion (TMVO) models in comparison with tMCAO models. Subsequent investigations suggested that the harmful role of Tregs may be specific to the tMCAO model (Liesz et al., 2015). Supporting this notion, Wang et al. (2023b) observed that Treg depletion in tMCAO mice alleviated post-stroke renal fibrosis through the IL-10/glutathione peroxidase 4 (GPX4) signaling pathway, providing additional evidence for the potential deleterious effects of Tregs in the tMCAO model.

Tregs exhibit a dual role in AIS. On one hand, they protect neurons from inflammation-induced cell death, a function that is strongly supported by extensive research in permanent ischemia models. On the other hand, they may facilitate secondary microthrombosis, the final phase of the thromboinflammatory cascade, a phenomenon that is particularly pronounced in transient stroke models such as tMCAO and TMVO. Studies have indicated that the occurrence of thromboinflammation in TMVO models is influenced by laboratory-specific methodologies, particularly the severity of ischemia induced by filament insertion. Thus, thromboinflammation is not a universal feature of all ischemia-reperfusion models but rather a distinct characteristic of TMVO models (Gauberti and Vivien, 2015). Notably, secondary microthrombosis has not been observed in non-human primate models of ischemia-reperfusion (Gauberti et al., 2014). In human stroke patients, thromboinflammation is not universally present. Longitudinal MRI studies have reported minimal lesion expansion following successful recanalization after thrombolysis (Delgado-Mederos et al., 2007). However, approximately 18% of stroke patients undergoing endovascular reperfusion still develop delayed infarction (Kidwell et al., 2002). Overall, thromboinflammation is typically absent in non-traumatic MCAO stroke models and in the majority of stroke patients. Nevertheless, the TMVO model remains valuable, particularly given the lack of any experimental stroke model that has achieved consistent translational success in clinical practice (Veltkamp et al., 2015). Additionally, particular attention should be given to patients undergoing endovascular reperfusion therapy, since these patients remain susceptible to delayed infarction.

## Regulatory T Cells in Gut–Brain Crosstalk: Implications for Stroke-Induced Systemic Inflammation and Recovery

Stroke has been shown to significantly alter the composition of the gut microbiota (Yamashiro et al., 2017a, b). This shift is typically attributed to the dysregulation of the autonomic nervous system following a stroke, which results in reduced intestinal motility and impaired intestinal barrier function (Houlden et al., 2016). The gut microbiota influences immune responses by modulating the function of intestinal dendritic cells (DCs). These DCs, in turn, stimulate the production of IL-10-producing regulatory T cells (Tregs), which help improve stroke outcomes by suppressing interleukin-17-producing gamma-delta T cells (IL-17^+^ γδ T cells) in the gut. Notably, this process does not require Treg infiltration into the brain (Benakis et al., 2016).

**Figure 5:**
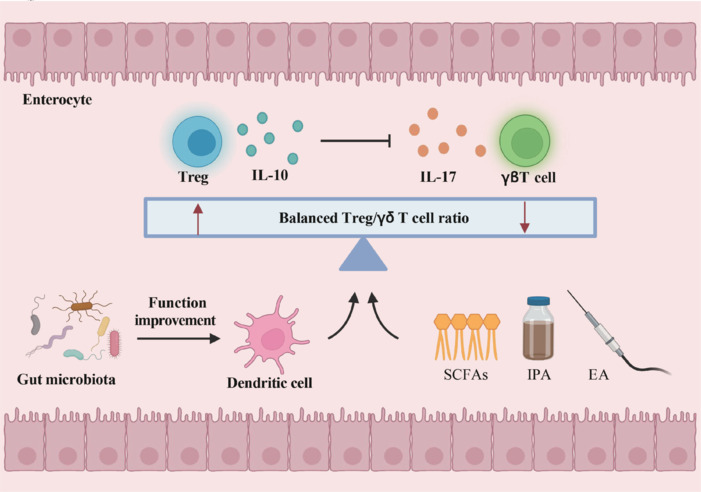
Tregs in the gut. The secretion of IL-10 by Treg cells can inhibit the secretion of IL-17 by γδ T cells and prevent their infiltration into the brain. The post-stroke intestinal microbiota can enhance the function of antigen-presenting cells, promoting Treg proliferation while inhibiting γδ T cell proliferation, thereby maintaining the Treg/γδ T cell balance. Certain therapeutic approaches, such as SCFAs, IPA, and EA, can also help to sustain this balance between Treg and γδ T cells. Created with BioRender.com. EA: Eicosapentaenoic acid; γδ T cells: gamma delta T cells; IL-10: interleukin-10; IL-17: interleukin-17; IPA: indole-3-propionic acid; SCFAs: short-chain fatty acids; Tregs: regulatory T cells.

In an experimental stroke model, a dysbiotic microbiota was shown to weaken the immune regulatory function of Tregs, leading to enhanced Th1 and Th17 pro-inflammatory responses in the gastrointestinal tract (Singh et al., 2016). These immune responses exacerbated brain injury by increasing the expression of the Th1 chemokine CCL5 (Dénes et al., 2010). Therefore, maintaining a balance between Treg and Th17 cells is critical for post-stroke recovery, since any disruption of this balance worsens neuroinflammation and brain damage (Wang et al., 2024a). For example, 2-(-2-benzofuranyl)-2-imidazoline, a potent neuroprotective agent, has shown the potential to restore the Th17/Treg balance in experimental stroke models. In rat models, 2-BFI significantly reduced infarct volume and neurological deficits while effectively inhibiting the proliferation of Th17 cells and the elevation of IL-17A levels. Additionally, 2-BFI increased the number of Tregs and promoted IL-10 expression in both brain tissue and peripheral blood. These findings suggest that regulating the Th17/Treg balance may provide a promising therapeutic strategy for stroke (Huang et al., 2020). Moreover, supplementation with short-chain fatty acids has been shown to effectively improve behavioral recovery in mice prior to stroke onset (Sadler et al., 2020). This improvement is correlated with a reduction in the proportion of IL-17^+^ γδ T cells in ischemic brain tissue (Lee et al., 2020). Similarly, indole-3-propionic acid has been found to maintain the integrity of the intestinal barrier and regulate Treg and Th17 cell functions, thereby contributing to stroke recovery (Xie et al., 2022b). Furthermore, electro–acupuncture has been shown to alleviate ischemia-reperfusion injury–induced intestinal inflammation in rats by modulating the polarization of Tregs and γδ T cells and enhancing intestinal barrier function (Wang et al., 2023a, b). In conclusion, these studies indicate the importance of maintaining a balanced Treg/γδ T cell ratio. This balance not only affects the gut-brain axis interactions but also regulates immune responses that influence the gut microbiota’s effect on ischemic brain injury (**Figure 5**).

However, the precise effects of different gut microbiota on stroke prognosis remain unclear. Notably, the presence of segmented filamentous bacteria in C57BL/6 mice from different commercial suppliers has been shown to correlate with variations in the Treg/Th17 cell ratio. In mice lacking segmented filamentous bacteria, treatment with CD28 superantigen (CD28SA) failed to induce Treg expansion, while mice from different suppliers exhibited varying experimental results (Sadler et al., 2017). Therefore, researchers should carefully compare the immune differences among various mouse strains when selecting models to ensure experimental reproducibility and reliability.

## Therapeutic Strategies for Modulating Regulatory T Cells in Stroke Recovery

### Adoptive transfer of regulatory T cells for stroke therapy

Adoptive transfer of Tregs is emerging as a promising immunotherapeutic strategy for modulating post-stroke immunity and promoting neuroprotection (Kleinschnitz et al., 2013). Currently, three primary approaches are used to expand freshly isolated Tregs, all of which rely on IL-2, a key regulator of Treg homeostasis and function. The first and most widely used strategy in experimental stroke models involves the *in vitro* expansion of polyclonal Tregs (pTregs). This process entails isolating Tregs from peripheral blood and then expanding them over a 2–3-week period using anti-cluster of differentiation 3/cluster of differentiation 28 (anti-CD3/CD28) magnetic bead stimulation in the presence of recombinant IL-2 and the mechanistic target of rapamycin (mTOR) inhibitor rapamycin, which prevents the proliferation of contaminating effector T cells (Fraser et al., 2018; Raffin et al., 2020). The second approach focuses on expanding antigen-specific Tregs, a technique commonly used in transplantation research. This method leverages donor-derived antigen-presenting cells, such as peripheral blood mononuclear cells (PBMCs), B cells, or dendritic cells (DCs), to stimulate recipient-derived Tregs, thereby inducing tolerance to donor antigens. While antigen-specific Tregs exhibit superior target specificity and reduced off-target immunosuppressive effects, their post-expansion yield remains limited, posing a challenge for large-scale applications. The third and most advanced strategy involves the genetic engineering of Tregs, where cells are modified to express synthetic receptors such as chimeric antigen receptors (CARs) or artificial T cell receptors, enabling precise antigen targeting. This method substantially enhances both Treg yield and immunomodulatory efficacy. CAR-Tregs have demonstrated therapeutic potential in models of transplant rejection (MacDonald et al., 2016; Boardman et al., 2017) and experimental autoimmune disease (Tenspolde et al., 2019). Recent advancements have led to the development of next-generation T cells redirected for universal cytokine-mediated killing, which secrete immunomodulatory cytokines upon CAR activation, further augmenting their therapeutic effects (Chmielewski and Abken, 2015).

In the early phase of stroke, the adoptive transfer of Tregs reduces MMP-9 levels, thereby preserving BBB integrity, limiting immune cell infiltration, and mitigating neurovascular damage (Li et al., 2013b). Additionally, Tregs modulate endothelial inflammation by suppressing CCL2 expression, which reduces perilesional edema and improves neurological outcomes (Mao et al., 2017a).

Beyond the acute phase, Treg therapy may contribute to neurorepair and functional recovery in the later stages of stroke. Studies have shown that the adoptive transfer of pTregs can mitigate chronic neuroinflammation and promote neural regeneration in both the acute (Liesz et al., 2009; Li et al., 2013a, c, 2014a; Brea et al., 2014) and chronic phases (Ito et al., 2019b; Shi et al., 2021). However, a major limitation is the extended *ex vivo* expansion time of 2–3 weeks, which may reduce the feasibility of using Treg therapy for AIS treatment. Nevertheless, research suggests that even delayed administration of expanded Tregs can enhance long-term recovery (Ito et al., 2019b; Shi et al., 2021). Future studies should explore whether late-stage adoptive Treg therapy can serve as a regenerative strategy for optimizing stroke outcomes.

Furthermore, Tregs exert potent anti-inflammatory effects following a stroke. Mao et al. (2017b) demonstrated that the adoptive transfer of Tregs inhibits the activation of the TLR4/NF-κB pathway, thereby reducing neuroinflammation. These findings suggest that Treg adoptive therapy may not only improve IS but also serve as a potential immunomodulatory strategy for hemorrhagic stroke.

### Strategies for expanding regulatory T cell populations

CD28 agonists, a class of monoclonal antibodies targeting the CD28 receptor on T cells, can promote the expansion and activation of Tregs through endogenous signaling pathways, thereby enhancing their immunosuppressive function (Badr et al., 2022). CD28 superagonists (CD28SA) have been shown to effectively expand Tregs and reduce neuroinflammatory responses via IL-10 secretion in IS models (Na et al., 2015). However, other studies have reported that CD28SA may exacerbate vascular lesions and trigger inflammatory thrombosis, potentially causing secondary infarction and ischemic neurodegeneration during the acute phase of experimental stroke (Schuhmann et al., 2015). Therefore, the safety of CD28 agonists in stroke therapy warrants further investigation.

IL-2 is a cytokine that induces T cell proliferation. The IL-2/IL-2 antibody complex selectively expands Treg populations by blocking IL-2 binding sites that are required for the proliferation of other T cell subsets (Shevach, 2012). Administration of IL-2/IL-2 antibody complexes, both prior to or following the onset of a stroke, has been shown to reduce infarct volume and improve neurological outcomes within 3 days post-MCAO (Zhang et al., 2018a). This effect is mediated through the activation of the CD39/CD73 signaling pathway, which enhances Treg proliferation and function. Additionally, Shi et al. (2021) reported that IL-2/IL-2 antibody complexes can increase Treg numbers and also enhance the reparative functions of microglia, thereby facilitating white matter repair and promoting neurological recovery. In *in vitro* stroke models, IL-2-treated Tregs have been shown to alleviate both gray and white matter damage by reducing TNF-α levels (Borlongan et al., 2021), although the improvements in white matter integrity may only become apparent during the later stages of stroke recovery (Yuan et al., 2023).

Despite promising preclinical evidence supporting IL-2-induced Treg expansion in mitigating white matter injury after IS, the clinical translation of these findings remains limited due to potential off-target immune activation and systemic adverse effects associated with IL-2 therapy (Hurst and Sohrabji, 2024). Therefore, additional studies are needed to address these limitations. Notably, the use of peripheral IL-2-mediated Treg expansion as a strategy for managing CNS inflammation may also induce systemic immunosuppression. To overcome this challenge, Yshii et al. (2022) demonstrated that direct delivery of IL-2 to the CNS can increase the population of brain-resident Tregs without compromising peripheral immune homeostasis.

IL-33 has been shown to increase Treg populations in both the ischemic brain and spleen following a stroke, indicating its neuroprotective role (Xiao et al., 2019). IL-33 exerts its effects by binding to its receptor, ST2 (IL-33R), promoting Treg proliferation and differentiation (Guo and Luo, 2020). This process is associated with downregulation of pro-apoptotic proteins and the secretion of Treg-associated cytokines (Xiao et al., 2019; Liu et al., 2020). Mechanistically, IL-33 induces Foxp3 expression in Tregs through ST2 signaling (Guo and Luo, 2020; Liu et al., 2020) and stimulates IL-2 production from CD11c^+^ dendritic cells (DCs), which selectively expand ST2^+^ Tregs (Matta et al., 2014). These Tregs secrete AREG, which activates the epidermal growth factor receptor on neurons, facilitating neurorepair and improving post-stroke prognosis (Guo and Luo, 2020). However, the anti-inflammatory properties of IL-33 may also increase susceptibility to infections. In experimental models, IL-33 administration exacerbated bacterial pneumonia following a stroke, worsening functional outcomes and increasing mortality within 24 hours. Combined treatment with antibiotics may help mitigate these systemic risks (Zhang et al., 2018b). Additionally, Liu et al. (2020a) demonstrated that IL-33 reduces neuronal apoptosis and enhances anti-inflammatory cytokine production through ST2-dependent Treg activation and expansion. IL-33 also strengthens Treg-mediated suppression of effector T cell proliferation (Xie et al., 2022a).

Histone/protein deacetylases (HDACs) modulate non-histone proteins, notably Foxp3, by inhibiting their DNA binding, which consequently reduces Treg numbers. In contrast, histone deacetylase inhibitors (HDACi) significantly increase Treg populations and enhance their immunosuppressive capacity. The neuroprotective effects of HDACi are critically dependent on Foxp3⁺ Tregs (Wang et al., 2018a). Mechanistically, HDACi induce IL-10 production from brain-infiltrating T cells, leading to enhanced phosphorylation of STAT3, a key transcription factor activated by IL-10. This mechanism suppresses pro-inflammatory cascades while upregulating neurotrophic factors essential for neuroprotection (Liesz et al., 2013). Furthermore, HDACi are potent inducers of Foxp3 expression, promoting the acetylation of Foxp3 and facilitating DNA binding (Huang et al., 2017). HDACi also promote microglial M2 polarization, reduce neuroinflammation and white matter damage, and confer neuroprotection in ICH models (Yang et al., 2021). The neuroprotective effects of HDACi have been shown to be dependent on Foxp3^+^ Tregs (Liesz et al., 2013). Additionally, Treg expansion mediated by HDACi may contribute to the attenuation of neuroinflammation following IS (Liesz et al., 2013).

Finally, in the chronic phase following a stroke, the administration of serotonin or selective serotonin reuptake inhibitors has been reported to promote the proliferation of brain-resident Tregs and improve neurological function. This effect is mediated through signaling pathways dependent on the 5-hydroxytryptamine recepto 7 (Ito et al., 2019a).

### Stem cell–based approaches to modulating regulatory T cell function

Stem cell therapy has emerged as a promising strategy for stroke recovery, primarily through Treg-mediated immune modulation and neural repair. Bone marrow–derived stem cells (BMSCs), which naturally contain Tregs, suppress pro-inflammatory cytokines and reduce secondary brain injury following a stroke (Neal et al., 2019). BMSC transplantation increases the proportion of Tregs, downregulates IL-6 and TNF-α expression, upregulates IL-10 expression, and improves neurological outcomes (Wang et al., 2014). Other stem cell types, including multipotent adult progenitor cells and embryonic stem cell-derived extracellular vesicles, also modulate immunity through Tregs. Multipotent adult progenitor cells restore post-stroke spleen mass, elevate splenic Treg levels and IL-10 levels, and reduce IL-1β levels, creating a reparative immune environment (Yang et al., 2017). Similarly, embryonic stem cell-derived extracellular vesicles significantly promote Treg proliferation and exert neuroprotective and immunoregulatory effects via Treg-dependent pathways (Xia et al., 2021).

Beyond immune regulation, stem cells promote neurorepair in a Treg-dependent manner. BMSCs facilitate the differentiation of OPCs into myelinating oligodendrocytes, thereby enhancing remyelination and white matter repair (Zarriello et al., 2019). Human induced pluripotent stem cells uniquely suppress post-stroke neuroinflammation and recruit Tregs to injured white matter, promoting remyelination and functional recovery (Xu et al., 2023). These findings highlight Treg recruitment as a critical mechanism for white matter repair, suggesting that targeting Treg mobilization may provide novel therapeutic avenues (**Additional Table 2**).

**Additional Table 2:** Studies on therapeutic strategies for expanding regulatory T cell populations in stroke

Study	Treatment method	Model	Treatment time point	Effects on brain infarct volume
Li et al., 2013a	Adoptive transfer	Mouse: 60 min tMCAO	2 h after tMCAO	Decreased
Li et al., 2013b	Adoptive transfer	Rat: 2 h tMCAO	2, 6, or 24 h after tMCAO	Decreased
	Adoptive transfer	Mouse: 60 min tMCAO	2, 6, or 24 h after tMCAO	Decreased
Kleinschnitz et al., 2013	Adoptive transfer	Mouse: 60 min tMCAO	24 h before tMCAO	Increased
Li et al., 2014a	Adoptive transfer	Mouse: 60 min tMCAO	2 h after tMCAO	Decreased
Brea et al., 2014	Adoptive transfer	Rat: tMCAO	2 h after tMCAO	Decreased
	CD28SA	Rat: tMCAO	4 d before tMCAO	Decreased
Na et al., 2015	CD28SA	Mouse: pMCAO	3 h after pMCAO	Decreased
	CD28SA	Mouse: 60 min tMCAO	3 h after tMCAO	Decreased
Schuhmann et al., 2015	CD28SA	Mouse: 60 min tMCAO	3 d before tMCAO or immediately after tMCAO	Increased
Zhang et al., 2018a	IL-2/IL-2 Ab	Mouse: 60 min tMCAO	3 d before, and 2 h after tMCAO	Decreased
Borlongan et al., 2021	IL-2/IL-2R	*In vitro* oxygen-glucose deprivation/reoxygenation	-	Decreased death of primary cortical or oligodendrocyte progenitor cells
Liu et al., 2020	IL-33	Mouse: 60 min tMCAO	0 h, 1 d, 2 d, and 3 d after tMCAO	Decreased
Guo and Luo, 2020b	IL-33	Mouse: 30 min tMCAO	24, 48, and 72 h of reperfusion	Decreased
Xiao et al., 2019	IL-33	Mouse: 30 min tMCAO	30 min before and 30 min after tMCAO	Decreased
Zhang et al., 2018b	IL-33	Mouse: 60 min tMCAO	24 h before and immediately after reperfusion	Decreased
Wang et al., 2014	BMSCs	Rat: pMCAO	24 h after pMCAO	Decreased
Liesz et al., 2013	HDACi	Mouse: pMCAO	Daily treatment starting 3 d before pMCAO	Decreased

This table summarizes studies investigating therapeutic strategies aimed at expanding or modulating Treg populations in experimental ischemic stroke models. The treatments examined include adoptive Treg transfer, CD28SA, IL-2, IL-33, and various pharmacological interventions. Treatment administration time points varied, ranging from pre-stroke induction to several hours post-stroke, and a variety of stroke models were used, including tMCAO and pMCAO. The effects on brain infarct volume were assessed, with the majority of studies showing a reduction in infarct size. However, some interventions, such as the pre-stroke administration of certain therapies, resulted in increased infarct volume. These findings highlight the potential of Treg-based and pharmacological therapies for stroke treatment, emphasizing that the timing and method of intervention are critical for efficacy. CD28SA: CD28 superagonists; IL-2: interleukin-2; IL-33: interleukin-33; pMCAO: permanent middle cerebral artery occlusion; tMCAO: transient middle cerebral artery occlusion.

Moreover, stem cells synergize with Tregs to enhance immunomodulation. Mesenchymal stem cells (MSCs) suppress activated T and NK cells (Gao et al., 2014), and the combination of MSCs with human-derived Treg therapy further reduces pro-inflammatory cytokine release while improving the brain’s reparative microenvironment (Yang et al., 2017). Similarly, NSC transplantation increases Treg populations while reducing pathogenic γδ T cells, thereby attenuating neuroinflammation (Gao et al., 2014).

However, the clinical translation of Treg-targeted stem cell therapy is limited by challenges in optimizing timing and dosing. BMSCs are most effective when administered within 3 to 24 hours post-stroke at doses ranging from 1 × 10^6^ to 1 × 10^7^ cells (Wang et al., 2014). Different stem cell types may act through distinct mechanisms at various phases of stroke; for example, BMSCs and human induced pluripotent stem cells primarily suppress inflammation in the acute phase while promoting remyelination in the chronic phase (Xu et al., 2023).

In summary, stem cell therapies hold substantial potential to improve stroke outcomes through Treg-mediated immune modulation and neuroregeneration. However, additional studies are required to elucidate the specific mechanisms underlying the effects of different stem cell types, optimize Treg-targeted recruitment, and ensure durable and precise therapeutic effects. Collectively, Treg-focused stem cell strategies may provide a novel and effective approach to enhancing recovery in both IS and ICH.

### Enhancing regulatory T cell stability and function for sustained neuroprotection

Studies describing the therapeutic strategies for enhancing Treg function in stroke are summarized in **Additional Table 3**. Mucosal immunity has emerged as a promising approach to induce cerebrovascular-specific expansion of Tregs, which play a pivotal role in modulating post-stroke inflammation and facilitating neural repair. Evidence suggests that weaker antigenic stimuli preferentially activate Tregs rather than effector CD4^+^ T cells (Sakaguchi et al., 2008, 2010), providing a theoretical basis for Treg-mediated immune regulation following stroke. Notably, sensitization to brain antigens is associated with poor neurological outcomes (Becker et al., 2005), whereas induction of immune tolerance to brain antigens, such as myelin basic protein, reduces inflammation, limits the infarct size, and improves prognosis (Becker et al., 1997; Gee et al., 2008).

**Additional Table 3:** Therapeutic strategies for enhancing regulatory T cell function in stroke

Study	Treatment method	Model	Treatment time point	Effects on brain infarct volume
Chen et al., 2003	Mucosal immunization	Rat: pMCAO	Tolerized before pMCAO	Decreased
Gee et al., 2008	Mucosal immunization	Rat: 3 h tMCAO	Tolerized before tMCAO	Decreased
Becker et al., 2005	Mucosal immunization	Mouse: 60 min tMCAO	Immediately after tMCAO	Increased
Becker et al., 1997	Mucosal immunization	Rat: 3 h tMCAO	Tolerized before tMCAO	Decreased
Ishibashi et al., 2009	Mucosal immunization	Rat: pMCAO	Tolerized before pMCAO	Decreased
Xie et al., 2014	Rapamycininjected ICV	Rat: 60 min tMCAO	6 h post-stroke	Decreased

This table summarizes studies aimed at enhancing the stability and function of Tregs for long-term neuroprotection following ischemic stroke. The interventions explored include mucosal immunization, pharmacological treatments, and immune modulation techniques designed to promote Treg-mediated neuroprotection. Animal models involved various durations of MCAO, with treatments administered either prior to or following stroke onset. The studies consistently report a reduction in brain infarct volume, indicating that therapeutic strategies targeting Treg function can mitigate secondary neuronal injury and improve stroke outcomes. The timing and specific approach to treatment were crucial factors influencing the degree of infarct reduction, underscoring the potential of Treg modulation as a therapeutic avenue for stroke recovery. ICV: Intracerebroventricular; MCAO: middle cerebral artery occlusion; pMCAO: permanent middle cerebral artery occlusion; tMCAO: transient middle cerebral artery occlusion; Tregs: regulatory T cells.

Repeated low-dose mucosal tolerance, including intranasal administration of E-selectin, has been shown to expand Tregs and reduce the infarct size after stroke (Chen et al., 2003). E-selectin-specific Tregs enhance Treg accumulation in ischemic brain tissue and cervical lymph nodes, suppress vascular TNF-α expression, and increase the levels of doublecortin, thereby supporting neuronal survival and functional recovery (Ishibashi et al., 2009). Additionally, CCL17, a key chemokine for Treg migration, facilitates Treg recruitment into the brain and promotes M2 polarization of microglia/macrophages, mitigating inflammation and injury following ICH (Deng et al., 2023).

Beyond mucosal tolerance, pharmacological agents such as rapamycin further enhance Treg function. Early administration of rapamycin post-stroke has been shown to reduce infarct volume, improve motor recovery, and suppress γδ T cell and neutrophil infiltration while promoting Treg expansion and upregulating the expression of Foxp3, CD25, and TGF-β1/Ebi3, thereby bolstering their anti-inflammatory capacity (Collison et al., 2007; Xie et al., 2014). Similarly, in ICH models, rapamycin increases Treg numbers and elevates IL-10 and TGF-β levels in both peripheral and brain compartments, yielding improved neurological function (Lu et al., 2014). Furthermore, PD-L1 administration decreases Th1 and Th17 cell populations while increasing Th2 and Treg levels, leading to reduced inflammation and preserved BBB integrity post-ICH (Han et al., 2017). Additionally, fingolimod enhances Treg populations in both peripheral and brain tissues by inhibiting S1PR-1-mediated lymphocyte trafficking, which boosts Treg immunosuppressive functions and reduces brain injury (Malone et al., 2021, 2023).

In summary, mucosal immune tolerance strategies (e.g., administration of E-selectin and CCL17) and pharmacological enhancers (e.g., rapamycin, PD-L1, and fingolimod) effectively promote Treg expansion and activation, representing a Treg-centered immunotherapy approach for both ischemic and hemorrhagic strokes. These insights underscore Treg modulation as a critical target for the development of future stroke therapies.

## Translational and Experimental Considerations for Regulatory T Cell–Based Therapies in Stroke

### Insights from preclinical stroke models and their limitations

IS models exhibit significant variability, posing unique challenges in studying the role of Tregs. For instance, the distal permanent occlusion model of IS induces a more robust neuroinflammatory response (Zhou et al., 2013), resulting in a notably higher number of T cells within the ischemic hemisphere than in the proximal temporary occlusion model (Liesz and Kleinschnitz, 2016). During the first week following transient ischemia, only a small population of CD25^+^Foxp3^+^ Tregs is observed (Gelderblom et al., 2009). In contrast, in the distal permanent MCAO model, Tregs constitute approximately 20% of all CD4^+^ T cells (Liesz et al., 2009; Llovera et al., 2014). Additionally, the cerebellar TMVO model induces a significant thromboinflammatory response within the first 24 hours post-stroke—an effect that is absent in the permanent occlusion model (Gauberti and Vivien, 2015). Remarkably, the proportion of Tregs relative to CD4^+^ T cells fluctuates significantly during the progression of both small- and large-volume strokes (Pang and Qian, 2017), indicating a possible link between stroke severity and the immunological function of Tregs. Liesz et al. (2015) indicated that increasing the quantity or activity of Tregs in models of mild to moderate injury may yield favorable immunological outcomes.

Despite advancements in IS models, mouse models of brain hemorrhage still show several limitations. In particular, due to the spontaneous recovery of motor function in rodents and the limited evidence of cognitive deficits (Zille et al., 2022), these models do not fully replicate the complex etiology of human spontaneous ICH. While current mouse models reflect certain pathological features of ICH, they do not entirely mirror the clinical presentation of the human condition. Despite the species differences in Treg numbers, studies conducted on larger-brained species may help address some of the discrepancies between models. For instance, Treg therapies have been utilized in pig and non-human primate models in fields such as organ transplantation, autoimmune disease treatment, and graft-*versus*-host disease suppression (Uehlein et al., 2021). Clinical trials have demonstrated that Treg therapies exhibit both safety and potential therapeutic efficacy (Di Ianni et al., 2011; Bluestone et al., 2015).

Finally, the influence of Treg-depletion methods on experimental results also requires consideration. For example, depleting Tregs using anti-CD25 antibodies (Liesz et al., 2009, 2013; Li et al., 2013b; Stubbe et al., 2013; Xie et al., 2014) typically leads to poorer prognoses, whereas depleting Tregs with diphtheria toxin in Foxp3 dTR mice (Ren et al., 2010) does not significantly influence the results. A crucial issue is that these depletion strategies affect both peripheral and brain-resident Tregs, potentially compromising the accuracy of the findings. Tregs can selectively target specific inflammatory responses rather than exerting systemic effects. This selective action may help explain why some immune-based therapies fail to achieve the expected results (Planas and Chamorro, 2009). Therefore, more precise Treg-depletion methods are essential to accurately investigate the functional differences between peripheral and brain-specific Tregs (Liston et al., 2022). A new gene delivery system has been developed to specifically increase IL-2 expression within the CNS, facilitating targeted investigations into the effects of distinct Treg populations (**Additional Table 4**).

**Additional Table 4:** Comparison of results from different T cell depletion methods

Study	Model	Lesion (%hemisphere)	Latest endpoint	Depletion model	Depletion efficacy	Effects on brain infarct volume
Liesz et al., 2009	pMCAO	≈10%	7 d	(1) Anti-CD25; (2) Adoptive cell transfer	90% n.a.	Increased
Liesz et al., 2013	pMCAO	≈10%	7 d	Foxp3-KO	85%	Increased
	30 min tMCAO	≈10%	7 d	Anti-CD25	90%	Increased
Xie et al., 2014	90 min tMCAO	≈40%	3 d	Anti-CD25	65%	Increased
Liesz et al., 2009	90 min tMCAO	≈55%	7 d	Anti-CD25	90%	No effect
Li et al., 2013b	60 min tMCAO	≈50%	3 d	Anti-CD25	90%	No effect
Stubbe et al., 2013	30 min tMCAO	≈50%	27 d	Anti-CD25	75%	No effect
Liesz et al., 2013	60 min tMCAO	≈50%	3 d	Foxp3-KO	85%	No effect

This table compares the effects of various T cell depletion methods on brain infarct volume in ischemic stroke models. The studies listed used different depletion strategies (e.g., anti-CD25, adoptive cell transfer, Foxp3 knockout) and evaluated their impact on lesion size (% hemisphere affected) at multiple time points post-stroke. The lesion size was measured at the latest endpoint for each study, ranging from 3 to 27 days post-stroke. In most studies, the anti-CD25 depletion strategy showed a significant increase in infarct volume, indicating that T cells play a protective role in ischemic injury. However, no effects on infarct volume were observed in some studies involving adoptive cell transfer or Foxp3 knockout models, suggesting differential roles of regulatory T cells in stroke pathology. Depletion efficacy was reported in terms of the percentage of depletion achieved by the respective treatments. The results provide valuable insights into the varied outcomes of T cell modulation in stroke, highlighting the complex role of immune regulation in brain injury and recovery. pMCAO: Permanent middle cerebral artery occlusion; tMCAO: transient middle cerebral artery occlusion.

### Challenges in clinical translation of regulatory T cell therapies

Although Treg therapy has demonstrated promising results in preclinical stroke studies, its clinical translation faces substantial challenges due to several issues, including immunosuppression, treatment toxicity, patient heterogeneity, optimal administration timing, and integration with existing therapeutic protocols. Although experimental models have shown Treg-mediated neuroprotection, several obstacles must be overcome before these therapies can be effectively applied in clinical settings.

The first challenge is the possibility that enhancing Tregs could potentially worsen post-stroke immune suppression, increasing susceptibility to infectious complications or cancer. However, animal studies have mitigated this concern, showing that Treg administration did not exacerbate immune suppression after stroke. Instead, it helped maintain lymphocyte populations in both the blood and spleen, thereby reducing the risk of post-stroke infections (Li et al., 2013a, b). Despite the absence of evidence from animal studies indicating that enhanced Tregs worsen immune suppression, long-term clinical data are still lacking. Therefore, additional clinical research and observations are needed to ensure that Treg therapy does not trigger additional immune-related adverse reactions in patients with compromised immune systems.

The second issue involves the potential adverse effects of Treg stimulants. Stroke patients often exhibit an imbalance in the Th17/Treg ratio, which is characterized by increased Th17 levels and decreased Treg levels (Hu et al., 2014; Dolati et al., 2018). This imbalance may lead to negative outcomes, such as depression (Swardfager et al., 2014) or fatigue (Liu et al., 2015). Enhancing Tregs may exacerbate this imbalance by promoting the conversion of Tregs into effector Th17 cells in the presence of IL-6 (Zheng et al., 2008). However, therapies that limit acetyl-CoA carboxylase 1 (ACC1) expression—such as caloric restriction or treatment with sorafenib analogs, microRNA-24-3p (miR-24-3p), or polyphyllin VII—can help correct this imbalance. These treatments reduce Th17 differentiation while increasing Treg populations, thereby decreasing neuroinflammation and improving stroke prognosis (Sang et al., 2024; Wang et al., 2019, 2024b). Moreover, targeting the receptor for advanced glycation end-products can modulate ACC1-dependent metabolic changes, reverse increased fatty acid synthesis in CD4^+^ T cells, and restore the Th17/Treg balance (Zhang et al., 2021b). Atorvastatin has also been shown to mitigate the negative effects of oxidized low-density lipoprotein on the Th17/Treg balance (Li et al., 2014b; Rodríguez-Perea et al., 2016). Therefore, combining Treg enhancement therapy with anti-ACC1 agents, soluble receptor for advanced glycation end products receptors, or atorvastatin treatment may be necessary. However, the potential adverse effects of Treg stimulants *in vivo* require thorough evaluation (Xia et al., 2016).

Beyond safety concerns, the clinical applicability of Treg therapy varies among patients with comorbidities. A previous study showed that Treg therapy enhanced by CD28SA can reduce infarct volume and promote neurological recovery in mice with type 2 diabetes mellitus, indicating potential benefits for patients with diabetic stroke (Cai et al., 2020). However, for patients with cancer, the situation is more complicated. The receptor for vascular endothelial growth factor, neuropilin-1 is highly expressed in Tregs. In tumor-bearing mice, a decrease in brain Treg levels was observed after stroke, while Tregs infiltrated tumors in large numbers, which could be attributed to increased vascular endothelial growth factor expression in tumor tissue. Therefore, inhibiting neuropilin-1 may be critical for Treg therapy in cancer patients experiencing stroke (Wang et al., 2018b). Further research is needed to determine the potential efficacy of Treg therapy in stroke patients with additional comorbidities. The timing of Treg administration is crucial in determining the therapeutic outcomes following stroke. Kleinschnitz et al. (2013) demonstrated that administering Treg therapy within 24 hours prior to the ischemic event resulted in suboptimal outcomes, suggesting that early Treg administration during the acute phase when the immune environment is still unstable, may produce detrimental effects. In contrast, a study by Ishibashi et al. (2009) found that pre-treatment with Tregs 10 days before ischemia significantly reduced infarct volume and improved stroke prognosis. This indicates that inducing Tregs earlier, before the stroke occurs, may allow the immune system to better adapt to the ischemic environment, thus providing protective effects. Following stroke, acute cerebral ischemia induces significant changes in the peripheral immune system, which are initially characterized by rapid immune activation (Offner et al., 2006a) followed by the onset of immune suppression syndrome (Meisel et al., 2005). Li et al. (2013a) showed that early Treg administration can alleviate the early inflammatory response and effectively prevent subsequent immune suppression, including lymphocytopenia. The biphasic role of Tregs—mitigating initial inflammation while preventing later immune dysfunction, particularly avoiding lymphocyte depletion and antigen-presenting cell exhaustion—is central to its therapeutic potential in stroke. Furthermore, early administration of Tregs has been shown to be significantly associated with a better prognosis 3 months after stroke. Conversely, patients with lower Treg levels within the first 2 days post-stroke are more prone to neurological deterioration and increased susceptibility to infections (Santamaría-Cadavid et al., 2020). These findings further suggest that rapidly enhancing Treg numbers may significantly improve long-term recovery. In conclusion, Treg tolerance therapy should be initiated at least 1 week before stroke and Treg therapy should be administered as soon as possible after stroke, typically within 1 week. The timing of Treg administration is crucial for influencing the outcomes of ischemic brain events, and additional studies are necessary to enhance treatment strategies (Hu et al., 2013).

### Age- and sex-related differences in Treg-mediated stroke outcomes

Although thymic Treg production and differentiation decline with age (Carpentier et al., 2013), paradoxically, the number of circulating and brain-resident Tregs significantly increases in older individuals in both mice (Raynor et al., 2012; Pasciuto et al., 2020) and humans (Zhao et al., 2007). This increase may be attributed to the enhanced survival of aged Tregs, which exhibit reduced expression of the pro-apoptotic gene Bim and upregulation of the anti-apoptotic gene Bcl-2, leading to greater resistance to apoptosis (Chougnet et al., 2011; Rocamora-Reverte et al., 2020).

The functional influence of aging on Tregs remains controversial, with studies reporting conflicting findings. Some studies have suggested that aged Tregs acquire enhanced immunosuppressive properties due to hypomethylation of cytosine-phosphate-guanine sites in the Foxp3 enhancer region, leading to increased Foxp3 expression and greater suppressive capacity than younger Tregs (Garg et al., 2014). Additionally, aged Tregs exhibit higher IL-10 expression and are more effective at inhibiting CD86 expression on dendritic cells than their younger counterparts (Hwang et al., 2009; Garg et al., 2014). Conversely, another study reported no significant functional differences between aged and young Tregs (Nishioka et al., 2006). Notably, aging appears to differentially affect distinct Treg functions. While aged Tregs demonstrate a reduced ability to suppress IL-2 and IL-17 production by non-Tregs, their capacity to inhibit antigen-presenting cell activation, T cell proliferation, and IFN-γ production remains comparable to that of young Tregs (Jagger et al., 2014).

Beyond global Treg function, aging may also influence post-stroke Treg responses. In young mice, the expression of cluster of differentiation 39 (CD39), a marker of functionally suppressive Tregs, remains stable following MCAO. However, in aged mice, CD39 expression declines in the peripheral blood after MCAO, indicating an age-related impairment in Treg-mediated immunosuppression following a stroke (Ruhnau et al., 2016). Despite these findings, the precise role of aging in post-stroke Treg function remains unclear, particularly due to the lack of studies using aged stroke models. Since most experimental research on this topic has been conducted in young animals, additional studies involving aged mice are essential to establish the clinical relevance of Treg-mediated protection in stroke.

Sex hormones exert significant immunomodulatory effects that influence both Treg function and stroke outcomes. Women aged 45–74 years typically exhibit smaller infarct volumes than age-matched men; however, this female-specific protection diminishes beyond 85 years of age, likely due to ovarian senescence and declining estrogen levels (Reeves et al., 2008). Estrogen has been shown to modulate Treg-mediated immune responses, providing neuroprotection following a stroke. In experimental stroke models, ovariectomized (OVX) adult rats demonstrate larger infarct volumes and more severe sensorimotor deficits than intact females. Interestingly, in reproductive-senescent OVX rats, these detrimental effects are attenuated, with improved post-stroke recovery and smaller infarct volumes. Further analysis revealed that reproductive-senescent -OVX rats have significantly higher levels of Tregs, M2-like macrophages, and MHC II^+^ cells in brain tissue than intact RS rats (Branyan et al., 2023). These findings suggest that Tregs, in conjunction with estrogen-driven immune modulation, contribute to sex-specific stroke protection.

Sex differences in stroke outcomes may also be influenced by spleen-dependent immune regulation. Studies have indicated that male mice with an intact spleen exhibit larger infarct volumes and fewer Tregs than female mice. However, this sex disparity disappears following splenectomy, resulting in comparable infarct volumes in both male and female mice. Further analysis revealed that splenectomy significantly reduces infarct volume in male mice but has no significant effect in females. Notably, splenectomy leads to a reduction in Treg numbers in female mice but not in males (Dotson et al., 2015). Thus, estrogen may regulate Tregs via the spleen, potentially mediated by regulatory B10 cells (Seifert et al., 2017).

Afeer stroke, female mice exhibit an increase in total spleen cell count but a reduction in B10 cell levels, indicating that B10 cells may migrate to the injured brain. Indeed, a previous study has reported a significant increase in B10 B regulatory cells (Bregs) in the ipsilateral hemisphere of female mice, but not in males (Seifert et al., 2012). The presence of B10 cells in the brain is thought to promote anti-inflammatory microglia and macrophages, thereby enhancing the neuroprotective response in female mice. Furthermore, Bregs can interact with T cells to enhance Treg function through the secretion of IL-10 and TGF-β (Yang et al., 2013). Thus, estrogen may contribute to increased counts of spleen cells, including B10 cells, which migrate to the brain and promote anti-inflammatory microglia/macrophages and Tregs through estrogen signaling and IL-10 production. However, splenectomy eliminates this advantage, leading to comparable infarct volumes in both sexes.

Conversely, androgens, particularly dihydrotestosterone (DHT), can modulate Treg-dependent immune responses following stroke. DHT has been shown to increase the proportion of Tregs within CD4^+^ T cells in orchidectomized male mice post-MCAO, but this effect has minimal influence on the early immune responses in the CNS. Notably, DHT replacement in castrated male mice results in significantly smaller infarct volumes at 96 hours post-MCAO, potentially by altering the functions of infiltrating immune cells and reducing their pro-inflammatory activity (Dziennis et al., 2011). Although androgens are believed to exert neuroprotective effects in stroke, this protection is often counteracted by the post-stroke decline in testosterone levels observed in intact males. Additionally, the use of DHT as an androgen replacement is limited by its inability to replicate the natural diurnal fluctuations of male androgens (Lucas and Eleftheriou, 1980).

Sex hormones play a crucial role in regulating Treg-mediated immune responses and stroke outcomes. Estrogen enhances Treg function, contributing to neuroprotection post-stroke; the mechanisms underlying the effects of estrogen potentially involve spleen-derived B10 cells. In contrast, DHT increases the proportion of Tregs, potentially mitigating post-stroke inflammation and reducing infarct volume. However, the precise mechanisms underlying these effects remain poorly understood, and additional research to elucidate the regulation of Tregs by sex hormones may provide novel immunomodulatory strategies for stroke therapy.

### Recent advances in clinical translation

Despite the significant challenges facing the clinical application of Treg therapy, its potential as a therapeutic strategy for ischemic stroke remains an area of intense and promising investigation. Currently, the only FDA-approved reperfusion therapies for stroke are recombinant tissue plasminogen activator (rtPA) and mechanical thrombectomy (Phipps and Cronin, 2020). However, the emerging role of Tregs in enhancing the efficacy of these treatments is garnering increasing attention. In experimental mouse models, delayed administration of rtPA has been shown to disrupt the BBB and promote hemorrhagic transformation. In contrast, the immediate infusion of Tregs following rtPA treatment significantly mitigates this hemorrhagic transformation and enhances motor function. Mechanistic investigations reveal that Tregs exert their protective effects by inhibiting the upregulation of MMP9 and CCL2, thereby preserving the integrity of the BBB. Furthermore, Tregs have been shown to suppress CCL2 expression in endothelial cells, further enhancing their protective effects. These compelling findings suggest that the adoptive transfer of Tregs could not only enhance the safety but also the efficacy of rtPA therapy, offering substantial clinical benefits in the management of ischemic stroke (Mao et al., 2017a). Moreover, propofol, an anesthetic agent, has been found to promote Treg expansion while simultaneously suppressing the activity of inflammatory T cells, positioning it as an ideal anesthetic choice for use in reperfusion therapies (Wang et al., 2021). Recent studies also suggest that patients who experience hemorrhagic transformation after thrombosis exhibit lower Treg levels compared to those without hemorrhagic transformation, indicating that Treg levels may serve as a potential biomarker for predicting bleeding risk (Gong et al., 2021).

Beyond their therapeutic potential, Tregs may also serve as valuable prognostic biomarkers for stroke. Research has demonstrated that patients with higher ratios of CD4^+^ Tregs to lymphocytes (> 2.209) and CD8^+^ Tregs to lymphocytes (> 1.363) exhibit significantly lower National Institutes of Health Stroke Scale National Institutes of Health Stroke Scale and modified Rankin Scale scores both at admission and 3 months post-stroke (Li et al., 2021). Another study observed that variations in the Treg/CD4^+^ lymphocyte ratio correlate with infarct size. In patients with larger infarcts, this ratio was markedly reduced, while in those with smaller infarcts, it remained relatively unchanged on the first day post-stroke (Pang and Qian, 2017). Intriguingly, irrespective of infarct size, the Treg/CD4^+^ ratio tends to increase in the later stages of stroke, suggesting its potential as a predictor of stroke severity (Pang and Qian, 2017). However, it is imperative to stratify and evaluate patients on an individual basis to tailor the most appropriate therapeutic approaches. The relationship between Treg levels and various clinical outcomes has also attracted considerable attention. Diminished peripheral CD4^+^ Treg ratios, coupled with elevated IL-6 levels, have been linked to a decline in executive function in patients with vascular cognitive impairment (Guoping et al., 2015). Furthermore, Treg levels below 0.027 cells have been identified as independent predictors of major adverse cardiovascular and cerebrovascular events during the perioperative period (Scholz et al., 2020). Nonetheless, baseline Treg levels have not been shown to correlate significantly with the development of acute coronary events or stroke (Wigren et al., 2012).

Recent advances in optical imaging, nuclear imaging, and magnetic resonance imaging technologies have paved the way for the *in vivo* visualization of Tregs. Notably, Tremblay et al. (2018) showed that Tregs labeled with superparamagnetic iron oxide particles can be tracked using magnetic resonance imaging. Furthermore, the combination of single photon emission computed tomography/positron emission tomography with computed tomography has been shown to enhance localization accuracy (Sharif-Paghaleh et al., 2011). These breakthroughs open new avenues for the clinical application of Tregs in diagnostic settings.

While significant challenges remain—including the complexities of immune regulation, patient variability, and discrepancies between preclinical and clinical outcomes—the expanding body of research suggests that Tregs could emerge not only as a promising therapeutic option but also as key biomarkers for stroke prognosis. As this field advances, Treg therapy is poised to revolutionize the landscape of stroke treatment and other neuroinflammatory disorders.

## Limitations

This review systematically investigates the multifaceted roles of Tregs in stroke, from acute neuroprotection to chronic neurorepair. However, several limitations remain. First, current research focuses mainly on neuroinflammation after cerebral ischemia, with limited studies on cerebral hemorrhage. Although commonalities between ischemic and hemorrhagic stroke have been noted, their distinct mechanisms are insufficiently addressed. Second, most evidence is based on rodent models, which cannot fully reflect the complex pathophysiology of human stroke. Clinical studies are scarce, and patient heterogeneity in age, sex, comorbidities, and treatments poses major challenges for translating preclinical findings. Third, while various strategies to enhance Treg function—such as adoptive transfer, drug-induced expansion, and immune modulation—have been proposed, their long-term safety and efficacy remain unproven and require validation in clinical trials. Finally, the interactions between Tregs and other immune and neural cells during stroke recovery are not fully understood, particularly within the gut-brain axis and systemic immune responses

## Conclusion and Future Perspectives

**Figure 6:**
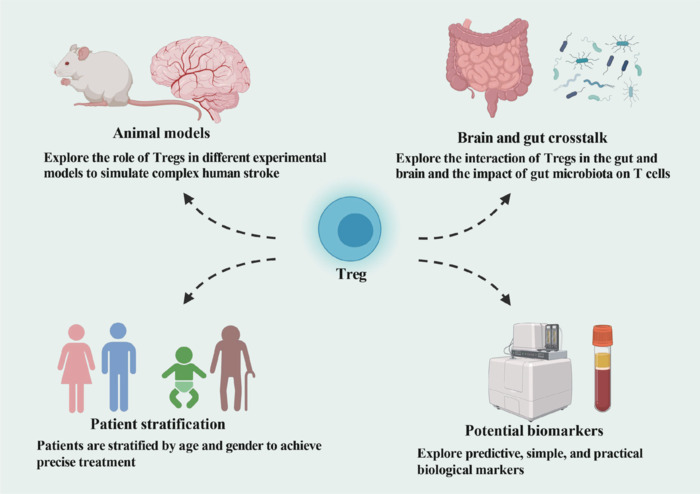
Prospective research directions for regulatory T cells (Tregs). There are four main aspects related to Tregs. First, we can investigate their multifaceted involvement across diverse experimental models to replicate the complex nature of human stroke pathology. Second, we can examine the intricate interplay of Tregs within the gut–brain axis and evaluate how gut microbiota influences Treg function and modulation. Third, we can analyze the most effective timing and strategies for therapeutic interventions, tailoring treatments based on sex- and age-specific considerations. Finally, we can investigate the utility of predictive, user-friendly biological markers to enhance clinical outcomes. Created with BioRender.com.

As pivotal regulators of post-stroke immunity, Tregs play multifaceted roles in modulating neuroinflammation and promoting tissue repair, offering great potential as therapeutic targets. Through dynamic interactions with inflammatory cells both peripherally and within the brain, Tregs orchestrate critical immune responses that shape stroke outcomes. However, significant challenges remain in translating Treg-based therapies to clinical practice. Future research should focus on delineating Treg functions across diverse experimental models to better capture the complex pathophysiology of human stroke. In addition, individual factors such as gut microbiota composition, age, and sex markedly influence Treg function and therapeutic efficacy, highlighting the need for personalized treatment strategies. Notably, Tregs are also involved in standard recanalization therapies, including rtPA and mechanical thrombectomy, where they mitigate blood-brain barrier disruption and modulate inflammatory responses, suggesting an underexplored role in optimizing current stroke interventions. Moreover, Tregs show promise as biomarkers for stroke severity and prognosis, further indicating their clinical relevance. Although substantial hurdles remain, continued research on Treg biology will be essential to unlocking their full therapeutic potential, ultimately advancing stroke treatment and prognosis (**Figure 6**).

## Additional files:

***Additional Table 1:*** *Search strategy for PubMed database.*

***Additional Table 2:*** *Studies on therapeutic strategies for expanding regulatory T cell populations in stroke.*

***Additional Table 3:*** *Therapeutic strategies for enhancing regulatory T cell function in stroke.*

***Additional Table 4:*** *Comparison of results from different T cell depletion methods.*

## Data Availability

*All relevant data are within the paper and its Additional files*.
